# Multiple Enol–Keto Isomerization and Excited-State Unidirectional Intramolecular Proton Transfer Generate Intense, Narrowband Red OLEDs

**DOI:** 10.1021/jacs.4c07364

**Published:** 2024-08-23

**Authors:** Xiugang Wu, Chih-Hsing Wang, Songqian Ni, Chi-Chi Wu, Yan-Ding Lin, Hao-Ting Qu, Zong-Hsien Wu, Denghui Liu, Ming-Zhou Yang, Shi-Jian Su, Weiguo Zhu, Kai Chen, Zi-Cheng Jiang, Shang-Da Yang, Wen-Yi Hung, Pi-Tai Chou

**Affiliations:** † School of Materials Science and Engineering, Jiangsu Engineering Laboratory of Light-Electricity-Heat Energy-Converting Materials and Applications, Changzhou University, Changzhou 213164, China; ‡ Department of Chemistry, National Taiwan University, Taipei 10617, Taiwan; § Department of Optoelectronics and Materials Technology, National Taiwan Ocean University, Keelung 20224, Taiwan; ∥ State Key Laboratory of Luminescent Materials and Devices and Institute of Polymer Optoelectronic Materials and Devices, South China University of Technology, Guangzhou 510640, China; ⊥ Robinson Research Institute, Faculty of Engineering, Victoria University of Wellington, Wellington 6012, New Zealand; # Institute of Photonics Technologies, National Tsing Hua University, Hsinchu 30013, Taiwan; ∇ Center for Emerging Material and Advanced Devices, National Taiwan University, Taipei 10617, Taiwan

## Abstract

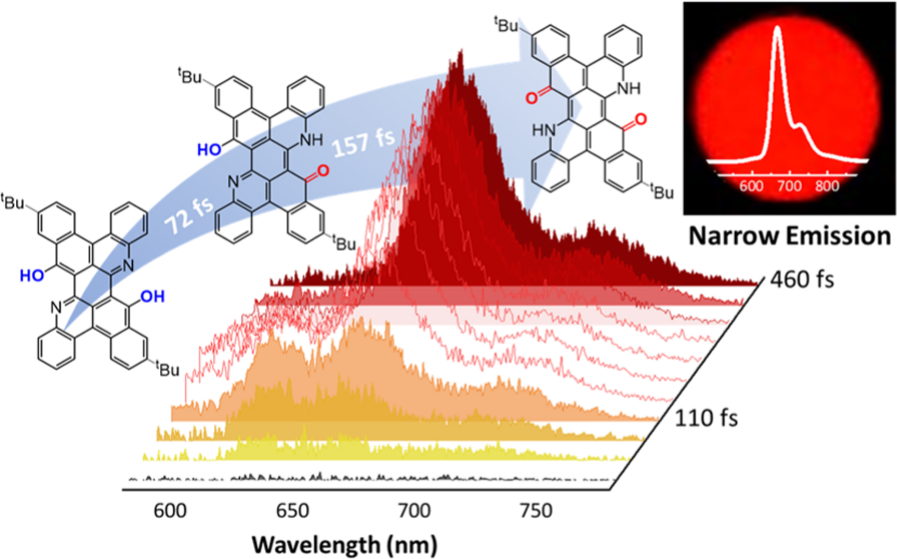

A novel series of excited-state intramolecular proton transfer (ESIPT) emitters, namely, **DPNA**, **DPNA-F**, and **DPNA-***^**t**^***Bu**, endowed with dual intramolecular hydrogen bonds, were designed and synthesized. In the condensed phase, **DPNAs** exhibit unmatched absorption and emission spectral features, where the minor 0–0 absorption peak becomes a major one in the emission. Detailed spectroscopic and dynamic approaches conclude fast ground-state equilibrium among enol–enol (EE), enol–keto (EK), and keto–keto (KK) isomers. The equilibrium ratio can be fine-tuned by varying the substitutions in **DPNA**s. Independent of isomers and excitation wavelength, ultrafast ESIPT takes place for all **DPNAs**, giving solely KK tautomer emission maximized at >650 nm. The spectral temporal evolution of ESIPT was resolved by a state-of-the-art technique, namely, the transient grating photoluminescence (TGPL), where the rate of EK* → KK* is measured to be (157 fs)^−1^ for **DPNA-***^**t**^***Bu**, while a stepwise process is resolved for EE* → EK* → KK*, with a rate of EE* → EK* of (72 fs)^−1^. For all **DPNAs**, the KK tautomer emission shows a narrowband emission with high photoluminescence quantum yields (PLQY, ∼62% for **DPNA** in toluene) in the red, offering advantages to fabricate deep-red organic light-emitting diodes (OLED). The resulting OLEDs give high external quantum efficiency with a spectral full width at half-maximum (FWHM) as narrow as ∼40 nm centered at 666–670 nm for **DPNAs**, fully satisfying the BT. 2020 standard. The unique ESIPT properties and highly intense tautomer emission with a small fwhm thus establish a benchmark for reaching red narrowband organic electroluminescence.

## Introduction

**Scheme 1:**
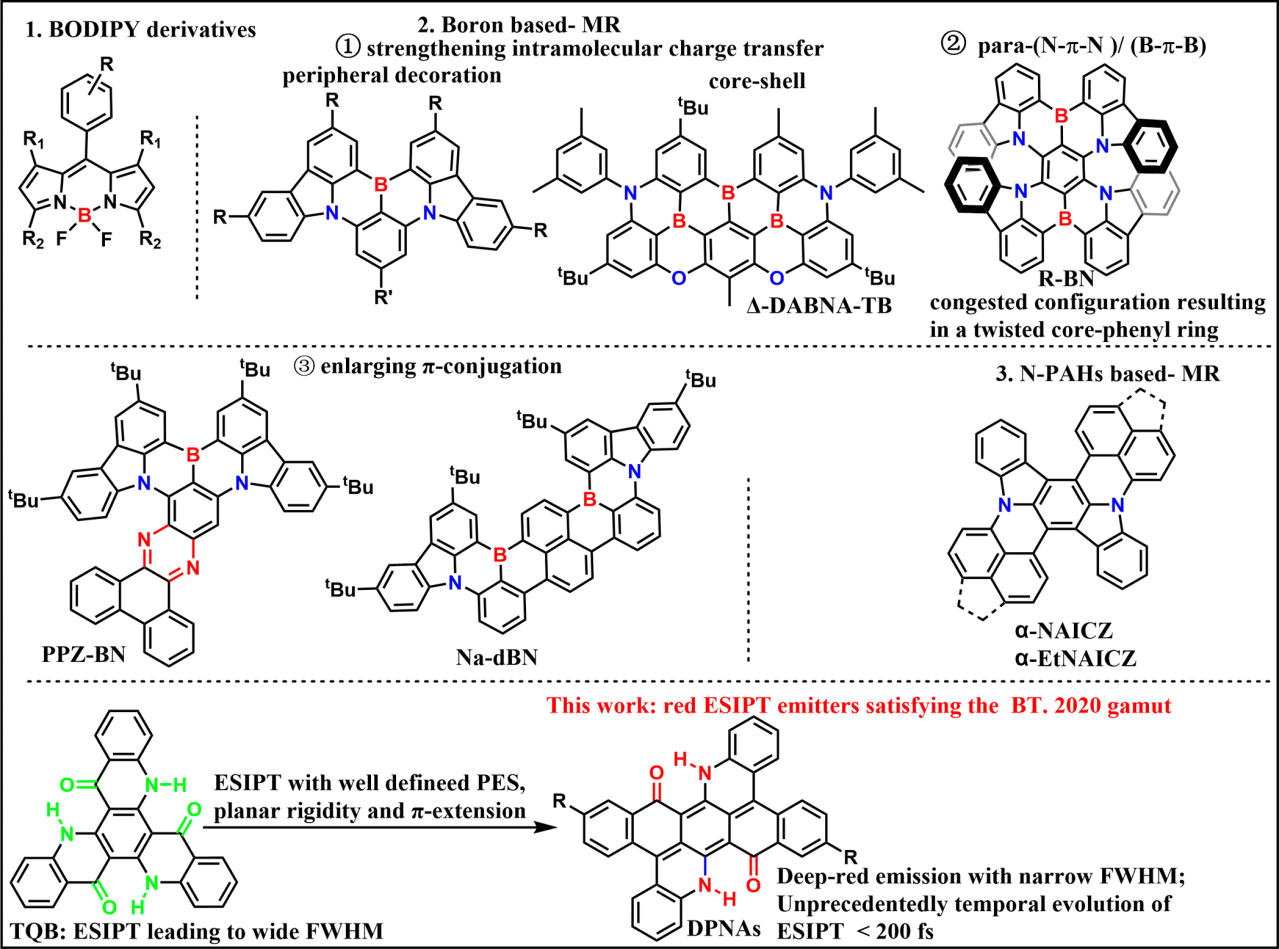
Some Representative Red Emitters with Narrow Bandwidth in OLEDs and the Molecular Design Principle for Proof-of-Concept ESIPT Emitters in This Study

Emitters with excited-state intramolecular proton transfer (ESIPT) properties are highly attractive because of their anomalously large Stokes-shifted proton transfer tautomer emission and spectral insensitivity to environment perturbation such as polarity and viscosity.^1^ Thanks to their unique emission spectral features, ESIPT emitters are particularly suitable for white light generation and two-color imaging applications.^2,3^ For instance, Park and co-workers proposed a “frustrated energy transfer” strategy to link blue- and orange-light-emitting ESIPT moieties in a white light generation.^4^ Our group successfully fine-tuned the energetics of ESIPT to equilibrium, achieving white light generation in a single ESIPT system.^5,6^ In addition to impressive white light generation, ESIPT emitters with single peak emission have also received attention. Yang et al. reported a series of nonrigid ESIPT molecules with a single green peak by integrating thermally activated delayed fluorescence (TADF) characteristics into an ESIPT core β-diketone to improve photoluminescence quantum yields (PLQY).^7^ You and co-workers utilized a series of ESIPT molecules based on 2-(2′-hydroxyphenyl)oxazole moiety to achieve deep-blue emission centered at 443 nm.^8^ Notably, Adachi and co-workers reported an ESIPT system, TQB (see Scheme 1), which also has TADF properties suitable for harvesting electroluminescence (EL) in organic light-emitting diodes (OLEDs) and emits green light.^9^

In yet another approach, ultrahigh-definition displays necessitate that OLEDs focus on color purity, particularly in meeting the high standards set by the Broadcast Television 2020 (BT. 2020) color gamut.^10^ One of the outcoupling methods to enhance color purity requires accessories such as color filters to achieve narrowband emission,^11^ which, however, leads to cumbersome device architecture, hence the reduction in both efficiency and cost-effectiveness. This shortcoming triggers the quest for the inherent narrowband emitters in attempts to remove outcoupled accessories. Furthermore, for the organic dye, it is also worth noting that attaining intense emission in the red to near-infrared (NIR) region presents a great challenge. The main hurdle lies in the increase of exciton-vibration coupling upon reducing the emission gap, resulting in the enhancement of the radiationless deactivation, the mechanism of which is often dubbed as the emission energy gap law.^12^ This, together with the Einstein spontaneous decay rate constant being inherently proportional to the third power of emission energy gap,^13^ leads to a significant decrease in the photoluminescence quantum yield (PLQY) toward red and near-infrared (NIR). Therefore, organic emitters with strong emissions and narrow bandwidth in the red or NIR have become a research focus of widespread concern. Currently, organic red emitters, particularly OLEDs based on hyperfluorescence technology (HF-OLEDs) present a promising solution for achieving high-efficiency red emission with a narrow bandwidth. For example, boron-dipyrromethene (BODIPY) derivatives in HF-OLEDs have achieved narrow emission bandwidth.^14−16^ Boron-based multiresonance (MR), with tactics such as peripheral decoration^17^ or core–shell^18^ strengthening intramolecular charge transfer, enlarging π-conjugation,^19,20^ and configuration with N–π–N and B–π–B emitters,^21−25^ represent another attempt to attain red emission with decent full width at half-maximum (FWHM) (Scheme 1). Additionally, nitrogen-atom-embedded polycyclic aromatic hydrocarbons (N-PAHs)-based MR emitters, namely, α-NAICZ and α-EtNAICZ, introduce homogeneous hexatomic rings to prolong the π-conjugation length, resulting in simultaneous red emission and a narrow FWHM.^26^ However, for the B/N emitters, by reducing the energy gap to the red, highest occupied molecular orbita/lowest unoccupied molecular orbital (HOMO/LUMO) separation is hindered, broadening the emission band.

To our knowledge, ESIPT molecules have paid almost no attention to red OLEDs, let alone their color purity. This rarity is attributed to the quite different potential energy surfaces (PES) between the tautomer ground state (S_0_) and excited state (S_1_), where S_1_ is commonly a bound state, while its corresponding S_0_ is usually unbound along the coordinates associated with proton relocation. This not only leads to the emission spectral broadening but also enhances the radiationless deactivation and hence low PLQY, particularly in the red and NIR.^27^

**Scheme 2:**
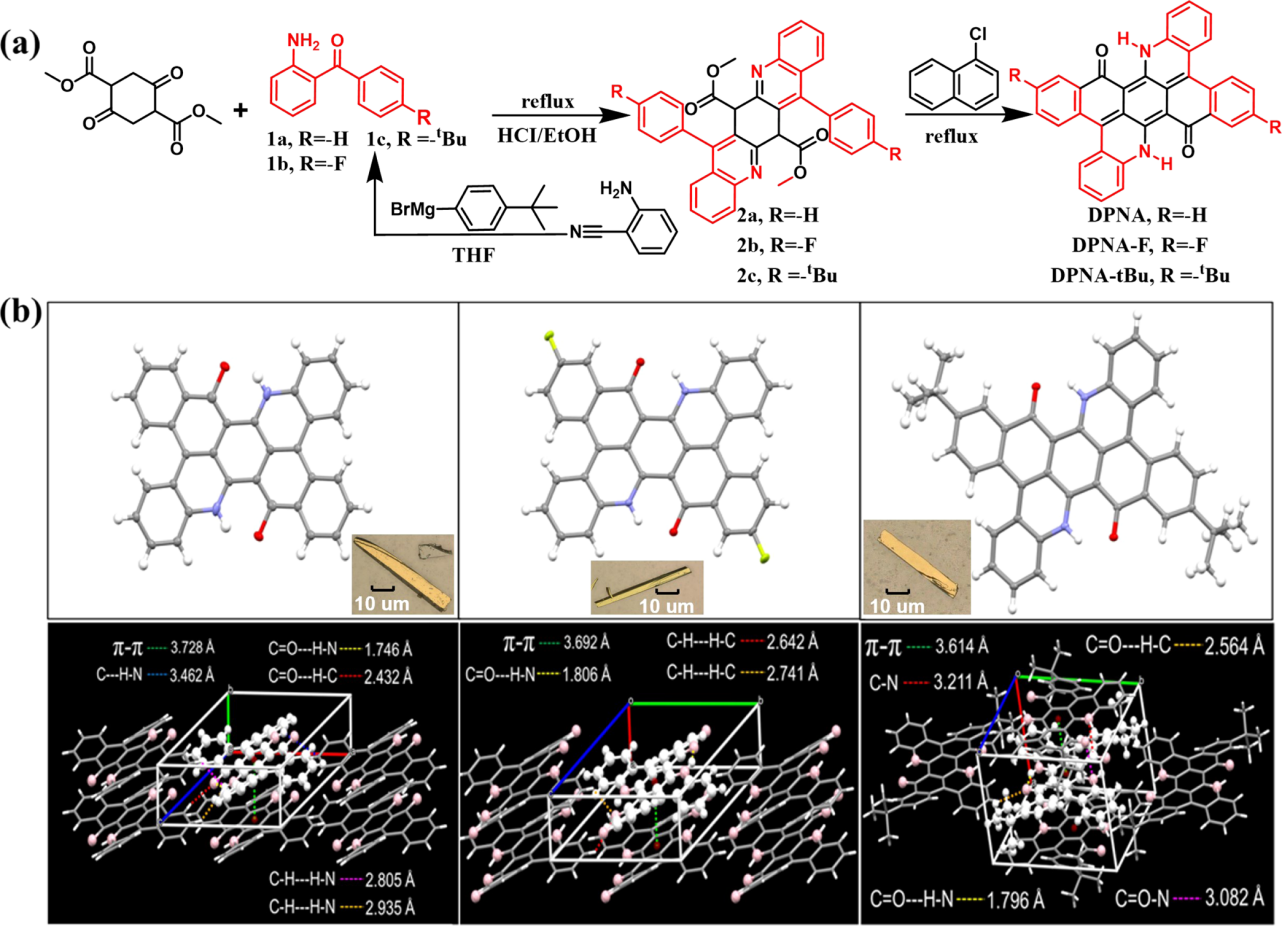
(a) Synthetic Route of **DPNA**, **DPNA-F**, and **DPNA-***^**t**^***Bu**; (b) (Top) Single-Crystal X-ray Structures of **DPNA**, **DPNA-F**, and **DPNA-***^**t**^***Bu**; the Insets Show the Optical Microscopy Images of Crystals by Vacuum Sublimation; (Bottom) Short Contact and H-Bond in Crystal Cell of **DPNA**, **DPNA-F**, and **DPNA-***^**t**^***Bu** For further structural data, refer to Table S1.

Herein, we propose a new strategy to address the aforementioned shortcomings of ESIPT molecules in red OLEDs. The de novo strategy is to incorporate rigid and planar π-conjugated ESIPT structures having well-bound PES for the tautomer ground state if there is any. The reduction of both spectral broadening and exciton–vibration coupling is thus expected, resulting in intense and narrowband tautomer emission in the red. Accordingly, a novel series of acridones-based ESIPT molecules, **DPNA**, **DPNA-F**, and **DPNA-***^**t**^***Bu** (Scheme 2) were designed and synthesized, which utilize a potentially N···H···O configuration to form dual intramolecular hydrogen bonds (H-bonds). The interplay of the dual H-bonds shows multiple enol-keto isomerization in the ground state and ultrafast proton transfer in the excited state. The spectral temporal evolution resolved by the state-of-the-art transient grating photoluminescence (TGPL) technique further unravels the mechanism of ultrafast ESIPT. The unidirectional ESIPT achieves intense, narrow FWHM (∼40 nm) tautomer emission with PLQY of >50% in the red (>650 nm), suitable for high-performance red OLEDs. The details of the results and discussion are elaborated in the following sections.

## Results and Discussion

### Synthesis and Characterization

Synthetic routes of **DPNA**, **DPNA-F**, and **DPNA-***^**t**^***Bu**, collectively referred to as **DPNAs**, are depicted in Scheme 2a, where detailed procedures are elaborated in the Supporting Information. In brief, shown in Scheme 2a, the corresponding intermediates of **DPNAs** are all obtained through a condensation reaction of dimethyl 2,5-dioxocyclohexane-1,4-dicarboxylate with aminobenzophenone (1) to yield (2), which is then heated in refluxing 1-chloronaphthalene. **1c** is obtained by the reaction of the prepared Grignard reagent and 2-aminobenzonitrile. The materials used for these reactions are extremely simple and cost-effective, and all have high yields, making them very promising for large-scale industrial production. All key intermediates were identified by ^1^H NMR and ^13^C NMR spectroscopy, as well as high-resolution mass spectrometry (HR-MS) (see Figures S2–S16).

Unfortunately, the synthesized **DPNAs** have sparse solubility in most organic solvents, even for **DPNA-***^**t**^***Bu** containing *tert*-butyl groups. The saturated concentrations of **DPNA**, **DPNA-F**, and **DPNA-***^**t**^***Bu** were measured to be 4.7 × 10^–4^, 2.8 × 10^–4^, and 6.8 × 10^–4^ M, respectively, in toluene, making the NMR measurement difficult. Alternatively, the structure is confirmed, rather than ever proposed one,^28^ by matrix-assisted laser desorption/ionization time-of-flight mass spectrometry (MALDI-TOF-MS), elemental analysis, and single-crystal X-ray diffraction (XRD) analyses, where, fortunately, single crystals of **DPNA**, **DPNA-F**, and **DPNA-***^**t**^***Bu** could be obtained by vacuum sublimation at sublimation temperatures of 350, 380, and 365 °C, respectively. The X-ray single-crystal analyses (Scheme 2b) indicate that all **DPNAs**, in common, show the presence of double H-bonds, e.g., 1.746 Å for **DPNA** along the N···H···O direction (Scheme 2b). However, in the X-ray analysis, the small H atom serves as a dummy atom for fitting of the X-ray structure. Therefore, it remains to be determined whether the H atom is bonded to N or O, forming a keto or enol configuration, respectively (vide infra). Moreover, the short contact of each planar framework induces intermolecular π–π stacking (Scheme 2b) with a π–π distance measured to be 3.728, 3.692, and 3.614 Å for **DPNA**, **DPNA-F**, and **DPNA-***^**t**^***Bu**, respectively, which correlates well with the increasing size of the peripheral substitution from hydrogen atom (**DPNA**), fluorine atom (**DPNA-F**) to the *tert*-butyl groups (**DPNA-***^**t**^***Bu**) in boosting the π–π stacking. Though decorated with *tert*-butyl groups, **DPNA-***^**t**^***Bu** still remains poorly soluble due to its dense π–π interaction hindering the insertion of solvent molecules. Moreover, their planar core-phenyl ring distinguishes them from the reported derivatives of twisted R-BN (see Scheme 1), which would facilitate the reduction of the reorganization energy, supported by the later spectroscopic measurement and computation simulation.

### Photophysical Properties

**Figure 1:**
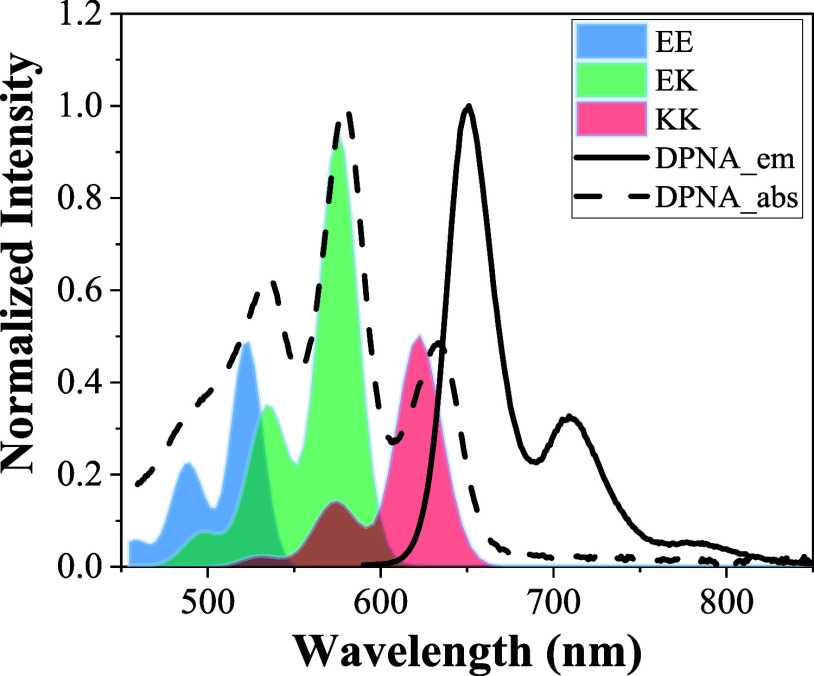
Absorption and emission spectra of **DPNA** in toluene (∼1 × 10^–5^ M). The colored-shaded data are the calculated absorption vibronic spectrum of KK, EK, and EE forms after optimization using the relative absorption coefficient as the fitting parameters for each isomer (see the text for details).

The absorption and emission spectra of **DPNA, DPNA-F**, and **DPNA-***^**t**^***Bu** in toluene are shown in Figures 1, S20a, and S20b, respectively. Pertinent data for the emission population lifetime and the associated PLQY are listed in Table S2. As shown in Figure 1, **DPNA** reveals a pronounced vibronic progression feature with three obvious peaks located at 535, 579, and 635 nm. Upon excitation at the absorption maximum, i.e., 579 nm, the emission band also shows prominent vibronic progression characterized by peak wavelengths at 651, 710, and 780 nm. Surprisingly, however, the relationship becomes anomalous because the absorption and emission spectral features are not mutually in a mirror image, which is unexpected from the Franck–Condon vertical transition for both absorption and emission. Instead, the 0–0 peak of the absorption that has minor intensity becomes the major peak in the emission. The excitation spectrum, being independent of the monitored emission wavelength, is identical, which is also the same as the absorption spectrum, inferring that the entire emission solely originates from the ground state, and there is no interference from any impurity. In other words, both the absorption and emission originate from **DPNA** inherently. Further support of this viewpoint is given by the similar spectral pattern in both steady-state absorption and their corresponding emission for **DPNA-F** and **DPNA-***^**t**^***Bu**, i.e., the obviously unmatched vibronic progression between absorption and emission (Figure S20 of the Supporting Information (SI)).

**Figure 2:**
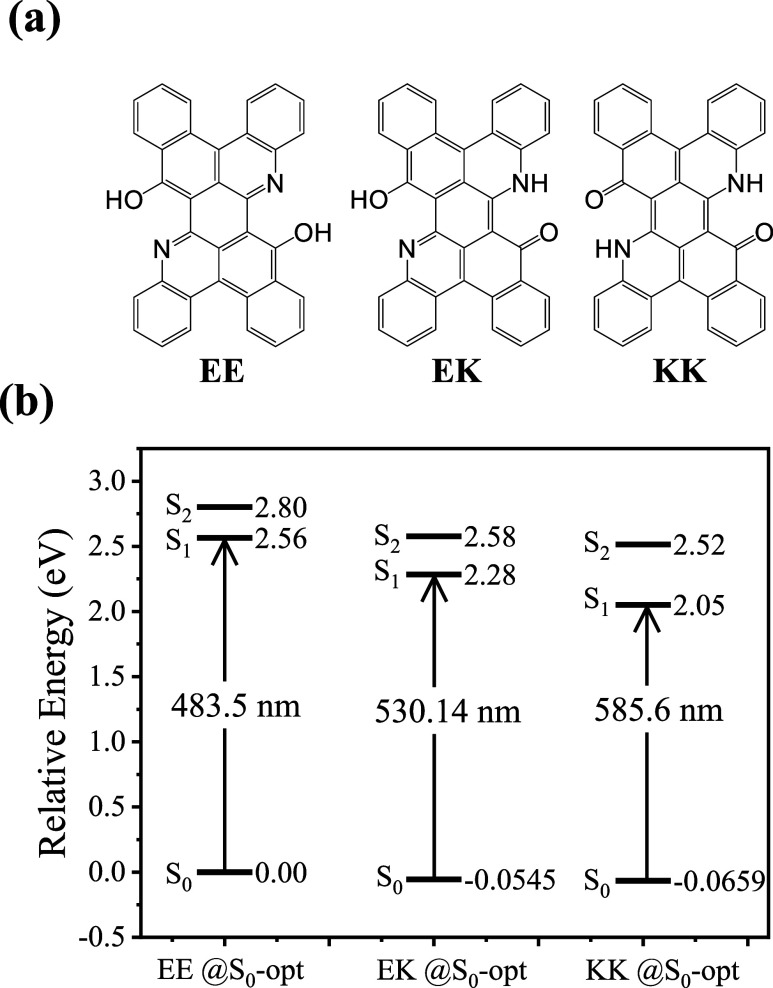
(a) Various isomers proposed for **DPNA**, enol–enol (EE), enol–keto (EK), and keto–keto (KK) isomers. (b) Calculated energy diagram (in eV) for KK, EK, and EE forms optimized at the ground state (S_0_) denoted by @S_0_-opt. Note: The calculation of the excited state (S_0_ and S_1_) here is based only on Franck–Condon vertical excitation to simulate the absorption spectra.

From the spectroscopy point of view, the results of non-mirror-imaged vibronic progression between absorption and emission may infer two possibilities. (i) It may indicate the existence of equilibrium among various isomers in the ground state, while independent of isomers, only one emitting species is observed. (ii) Alternatively, it could be the existence of a single species in the ground state, while equilibrium takes place among various isomers in the excited state, giving multiple emissions, the sum of which show different spectral features from that of the absorption. Considering that the N--H--O site is the only active center to account for the isomerization in **DPNA**, chemically, three types of proton transfer isomers can be drawn, which are categorized as enol–enol (EE), enol–keto (EK), and keto–keto (KK) isomers depicted in Figure 2a.

To address the two proposed mechanisms, the computational approach provides valuable information. Especially, the current computation capacity should be able to accurately estimate the ground-state thermodynamics of various isomers for a single molecule, such as **DPNA**. We thus carried out density functional theory (DFT) and time-dependent density functional theory (TDDFT) calculations for EE, EK, and KK isomers for **DPNA** in solutions such as toluene. Comprehensive computation was conducted at B3LYP/6-31g, wB97XD/6-31g, and B3LYP/6-31+g(d,p) levels (Gaussian 16 program) to gain an understanding of the correlation among the structural, thermodynamic, and optical properties. In brief, the B3LYP hybrid functional combines the local density approximation (LDA) with the generalized gradient approximation (GGA) and Hartree–Fock exchange, which is known for its good performance in predicting molecular geometries, vibrational frequencies, and thermodynamic properties. The long-range-corrected wB97XD functional, on the other hand, includes both Hartree–Fock exchange and second-order perturbation theory. In standard DFT methods, wB97XD also has a long-range correction term for the incorrect description of charge transfer and polarization effects. Overall, B3LYP is more accurate in predicting energies than wB97XD, and wB97XD is considered more accurate than B3LYP in the wave function description. However, wB97XD is also computationally more expensive. All pertinent data are listed in Table S3, with the associated frontier molecular orbitals of all **DPANs** shown in Figure S23.

Using **DPNA** as a paradigm, Figure 2b shows the calculated energy differences among KK, EK, and EE forms in the ground state and their relative absorption energies. Related to the EE form, EK and KK isomers are more stabilized by 0.0545 and 0.0659 eV, which, in terms of energy (kcal/mol) difference, is calculated to be 1.52 kcal/mol (EE) and 0.26 kcal/mol (EK) versus the lowest KK form set to be 0.0 kcal/mol in toluene. We then calculated the Boltzmann distribution for each form, giving the population ratio in the order of 0.044, 0.374, and 0.582 for EE, EK, and KK, respectively. To better understand the contribution of each species in the absorption spectra, we utilized TDDFT, including the Duschinsky and Herzberg–Teller (HT) effect, to simulate the absorption spectra, which are depicted in Figure S25. The vibrationally resolved optical spectra were computed at *T* = 0 K, including Franck–Condon contributions and a convoluted Gaussian with an FWHM = 350 cm^–1^. Also noticed is that all simulated spectra of EE, EK, and KK isomers for **DPNA** possess the strongest transition at the 0–0 peak at ∼520, 578, and 620 nm, respectively (see Figures 1 and S25). The results infer long π-delocalization and structural rigidity for all isomers; thus, a very small reorganization energy is expected for the S_1_ state (vide infra).

As for the spectral fitting, we simply treated the relative absorption coefficient to be the fitting parameters for each species and used their linear combination, i.e., ε_total_ = ε_KK_*c*_1_ + ε_EK_*c*_2_ + ε_EE_*c*_3_, where *c*_1_, *c*_2_, and *c*_3_ values are taken from the calculated Boltzmann population for KK, EK, and EE, respectively (vide supra). As a result, the experimental absorption spectrum, fitted using the calculated absorption for each form, is depicted in Figure 1. Upon optimization, the absorption coefficient ratio of EE/EK/KK was found to be 0.6:1:0.4 at the respective peak wavelength of each isomer. The asymmetrical structure of EK may lead to a relatively large transition dipole and hence an absorption coefficient.

**Figure 3:**
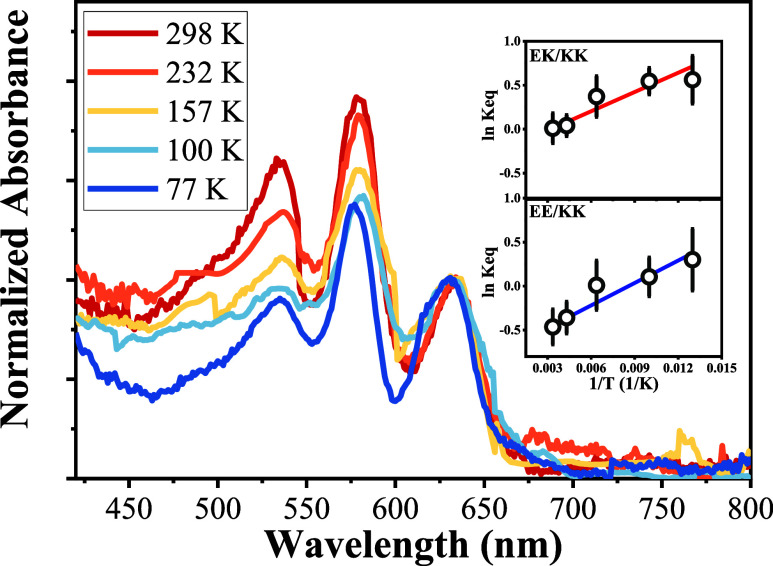
Absorption spectrum of **DPNA** in a methylcyclohexane/toluene (1:1 in volume) mixture under different temperatures. Inset: Van’t Hoff plot of ln *K*_eq_ versus 1/*T* at 530 and 578 nm, where *K*_eq_ is the equilibrium constant and *T* specifies temperature in Kelvin (K). Note that the spectrum was normalized at 633 nm to clearly present the absorbance evolution at various temperatures.

To further support the ground-state isomerism for **DPNAs**, experimentally, we conducted a temperature-dependent UV–vis absorption study of **DPNA**, where a methylcyclohexane/toluene mixture in a 1:1 volume ratio was used as the solvent to ensure the transparent glassy form at low temperatures (see the SI for experimental details). When the temperature decreases from 298 to 232 K (see Figure 3), although the ratio of peak absorbance at 635 and 579 nm changes slightly only, the 535 nm absorption band that is mainly ascribed to the EE isomer decreases significantly. This result thus shows that the EE isomer is more susceptible to the effect of temperature change, which is consistent with the calculated EE form being the highest in energy. Further decreasing the temperature from 157 to 77 K gives an obvious decrease of the 579 nm band, accompanied by a slight increase of the 635 nm band. This observation can be rationalized by the decrease in the EK population at 579 nm. However, the accompanying increase of the 635 nm absorption (KK form) does not seem to correlate well with the decrease of the EK isomer. One plausible explanation is due to the formation of the glassy matrix at low temperatures, resulting in the change of the dielectric constant, inducing spectral nonlinear correlation. Nevertheless, using the absorbance at 530 and 580 nm to represent the population of EE and EK forms (calibrated by the relative absorption coefficient of 0.6:1:0.4, vide supra), a Van’t Hoff plot gives a free-energy *G* of 0.65 and 0.28 kcal/mol for EE and KE above that of KK. Although this approach is primitive due to the uncertain changes of the temperature-dependent absorption coefficient and negligence of the spectral overlap among EE, EK, and KK, the result, in a qualitative manner, is consistent with the small energy difference estimated by the computational approach (vide supra).

Despite the small energy difference and hence thermal equilibrium of the three isomers in the ground state, in the excited S_1_ state, the energy difference between EE, EK, and KK is calculated to be significantly large for **DPNA**. As shown in Figure 2b, the geometry-optimized S_1_ state for the EE isomer is higher in energy than that of EK by 6.46 kcal/mol, and S_1_ of the EK isomer is higher than that of KK by 5.30 kcal/mol. Therefore, if proton transfer is kinetically allowed, regardless of which isomer is initially excited, the emission should originate from the KK* state (* denotes the electronically excited state). In other words, ESIPT is expected to take place from both EE* and EK* to the KK* isomer with corresponding emission. This prediction aligns with the steady-state measurement where the emission seems to originate from one species, i.e., the KK* form, showing a maximum at the 0–0 vibronic peak, which is mirror-imaged with that of the absorption profile calculated for the KK form but is much different from the absorption profile obtained experimentally (Figure 1). As shown in Figure S24 and Table S3, similar calculation results were obtained for **DPNA-F** and **DPNA-***^**t**^***Bu**, that is, the ground-state equilibrium among EE, EK, and KK forms at room temperature and thermally favorable ESIPT for EE* and EK*, resulting in solely the KK* isomer if ultrafast ESIPT kinetics take place.

### Transient Grating Photoluminescence to Probe Early Dynamics

A number of studies have investigated the ultrafast dynamics of keto–enol isomerization by employing femtosecond spectroscopy and soft X-ray spectroscopy.^29−32^ In an attempt to resolve the above-proposed fast ESIPT dynamics among various isomers, we carried out here a state-of-the-art experiment based on femtosecond transient grating photoluminescence (TGPL). We aim to probe the spectral temporal evolution of ESIPT among three isomers in the early relaxation dynamics expected to be within a hundred femtoseconds. TGPL is an advanced ultrafast photoluminescence method that enhances the signal contrast and accommodation bandwidth of spectral measurement by using a unique geometry to spatially separate the ultrafast broadband gated spectra from the background photoluminescence.^33^ This technique enables sensitive and high-time-resolution measurements, making it ideal for studying complex spectral dynamics. It is particularly valuable in photophysical studies of multichromophoric systems and materials with intricate energy or charge transfer processes.^34−36^ In brief, our home-built TGPL system started with a ytterbium laser (190 fs, 200 μJ, 1030 nm). The output pulse from the multiple-plate continuum system (50 fs, 160 μJ, 1030 nm) was split into pump (515 nm) and gate (1030 nm) pulses. The instrument response function (IRF) of the TGPL exhibits an 80 fs time resolution. Details of the experimental setup for TGPL and its layout are elaborated in the Supporting Information (see Figure S22 in the SI-TGPL section).

**Figure 4:**
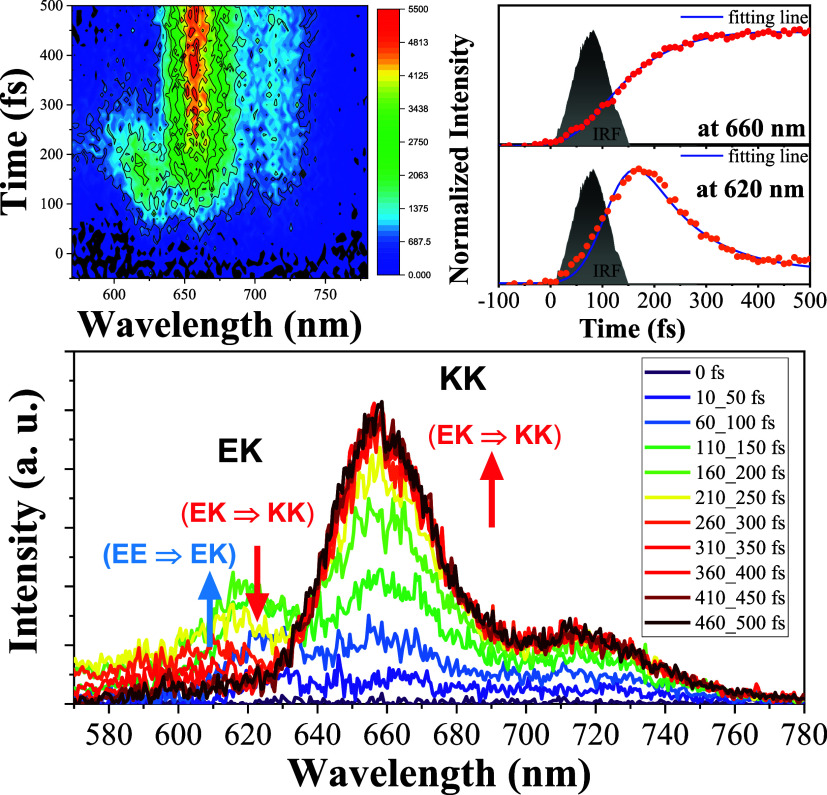
(Top left) TGPL evolution contour map. (Right) Relaxation kinetics at 655 nm (red) and 620 nm (orange) emission and instrument response factor IRF (gray). (Lower) Spectral temporal evolution of **DPNA-***^**t**^***Bu**. The sample is prepared in toluene. The excitation wavelength is 515 nm.

The low solubility of **DPNAs** in organic solvents (vide supra) makes any pump–probe experiments difficult, where TGPL is no exception. Given that **DPNA-***^**t**^***Bu** has the highest solubility and molar absorption coefficient (ε) among **DPNAs** in, e.g., toluene (see Table S2), we selected **DPNA-***^**t**^***Bu** to probe the evolution of transient emission. Luckily, the current 515 nm pulse is able to cover the excitation mainly at EE, and partially at EK and KK concurrently. Figure 4 (top left) displays an overall contour map detailing the spectral and temporal evolution. By extracting data at every 50 fs intervals, we captured the spectral and temporal changes depicted in Figure 4 (bottom), consisting of 620, 655, and 715 nm emission peaks. Specifically, for the 620 nm component, in a time range of 0–150 fs, we observed a growth of intensity, which reaches a maximum of around 200 fs, followed by a decrease of its intensity, and becomes virtually negligible at ∼360 fs. On the other hand, both 655 and 715 nm emission bands reveal a gradual increase of the intensity from *t* = 0, reaching a plateau around 300 fs and then remaining constant within an acquisition window of 500 fs. The 655 and 715 nm bands are identical with the 0–0 and 0–1 peaks in the steady-state emission ascribed to the KK isomer. We thus reasonably assign the 620 nm emission, which was not observed in the steady-state measurement, to the EK isomer. Note that this 620 nm 0–0 peak has an exact mirror image with the 590 nm absorption peak of the EK form according to the computation. Further plot of 655 nm intensity as a function of the pump–probe delay time (Figure 4 (top right)), gives a rise time of 157 fs. Taking this 157 fs as the decay time of the EK component (monitored at 620 nm), we obtained an ∼72 fs rise time for the 620 nm emission, as shown in Figure 4. Because the fitted 72 fs is shorter than the system response time of 80 fs, it is expected to have significant uncertainty.

**Figure 5:**
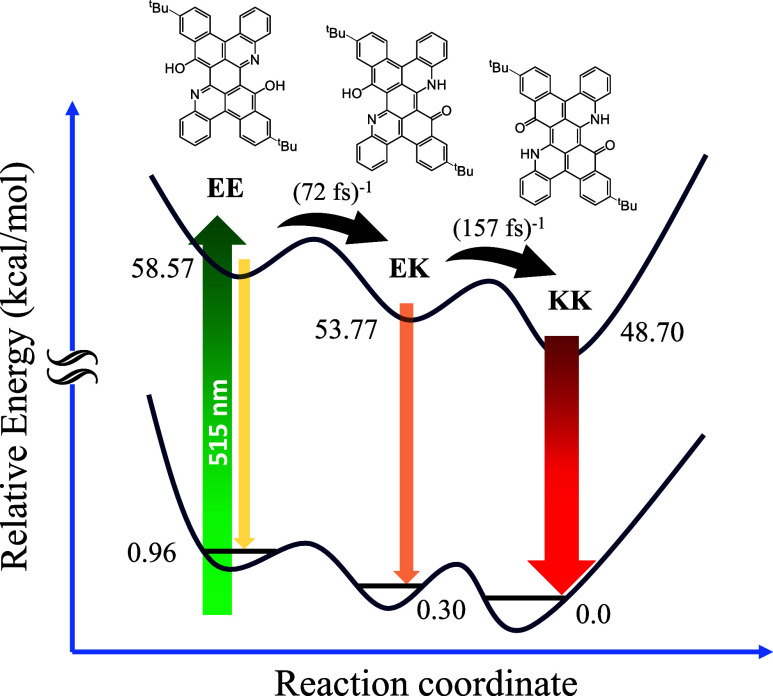
Proposed mechanism of ground-state isomerization and excited-state proton transfer reactions using **DPNA-***^**t**^***Bu** in toluene is shown as an illustration. Note: The unit of the numerical number is in kcal/mol.

Briefly, the results of TGPL lead to a clear ESIPT mechanism for **PANs-***^**t**^***Bu**, as shown in Figure 5, where despite a ground-state equilibrium between EE, EK, and KK, ultrafast ESIPT takes place in both EE* and EK*, resulting in KK* tautomer emission. For **DPANs-***^**t**^***Bu**, the rate of EK* → KK* is determined to be 157 fs^–1^, while a stepwise EE* → EK* → KK* double proton transfer takes place (see the SI for the kinetic expression), an EE* → EK* rate of ∼(72 fs)^−1^, followed by the EK* → KK* rate of (157 fs)^−1^. Note that the **DPNA-***^**t**^***Bu** contains only two *tert*-butyl groups (see Scheme 1 and Figure 5). This configuration suggests the possibility of two EK isomers differing in the mutual positions of the enol and keto sites toward the *tert*-butyl group. Since TGPL can only effectively resolve ESIPT-related chromophores, and the two EK isomers, in theory, are insensitive to *tert*-butyl groups, our current results cannot differentiate between the two EK isomers. Furthermore, we cannot draw conclusions about whether there will be a concerted EE to KK double proton transfer process, at least not from the TGPL data. Future sophisticated theoretical calculations may be able to address this question and provide a possible branching ratio for the two-step versus one-step ESIPT process.

### Device Fabrication and Performance

Unlike typical ESIPT molecules, where the tautomer emission in red is rather weak due to the unbound PES of the tautomer ground state along the proton-transfer-associated coordinates (vide supra), **DPNAs** possess a well-defined KK isomer, while the one and two proton transfer in the excited state takes place from EK* and EE* forms, respectively, giving solely KK* tautomer emission that simplifies the spectral profile. The well-defined KK state with a rigid, large π-delocalized structure gives intense red emission. In addition to the extended frontier orbitals, the MO distributions illustrated in Figure S23 show a certain extent of alternating distributions of HOMO and LUMO orbitals on the aromatic backbone, especially for the KK forms. This leads to reorganization energy calculated to be as small as 1.51–1.53 kcal/mol (0.065–0.068 eV, see Figure S26 with detailed elaboration in the SI), which experimentally reflects their high PLQY of 62.2, 52.3, and 72.8% in **DPNA**, **DPNA-F**, and **DPNA-***^**t**^***Bu**, respectively, in toluene (see Table S2). More importantly, the associated small reorganization energy enhances the 0–0 vibronic peak, narrowing the fwhm of the emission. These provide all of the advantages worth pursuing for OLED applications.

**Figure 6:**
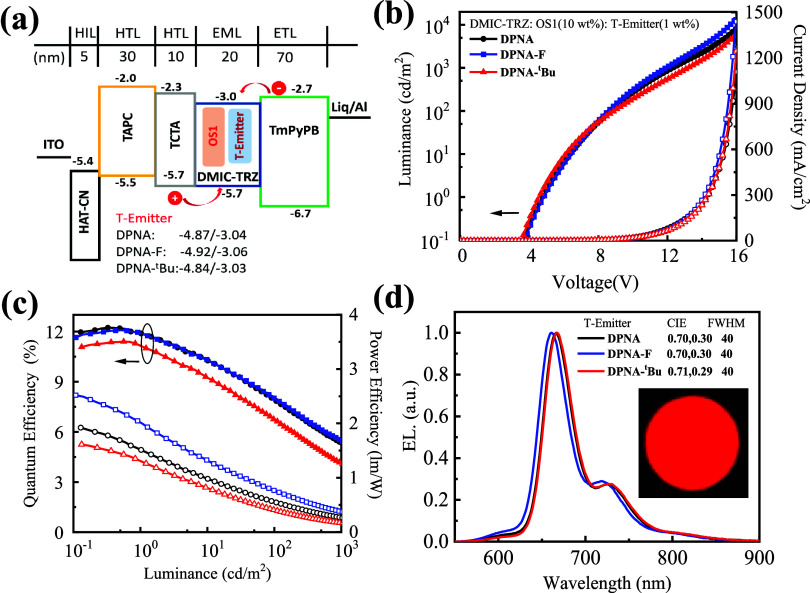
(a) Energy diagram of hyper-OLED devices fabricated in this study, (b) current density–voltage–luminance (*J*–*V*–*L*) characteristics, (c) respective external quantum efficiency (EQE) and power efficiency (PE) diagrams as a function of luminance, and (d) EL spectra of devices fabricated using **DPNA**, **DPNA-F**, and **DPNA-***^**t**^***Bu** as terminal emitters.

In examining electroluminescence (EL) characteristics, we selected **DPNA**, **DPNA-F**, and **DPNA-***^**t**^***Bu** as the terminal emitters for constructing hyper-OLED devices. Hyper-OLEDs offer the additional capability of enhancing efficiency by channeling energy from sensitizers to fluorescent emitters.^37,38^ To evaluate their EL properties, OLEDs were fabricated employing the following optimized device configuration: indium tin oxide (ITO)/HAT-CN (5 nm)/TAPC (30 nm)/TCTA (10 nm)/EML (20 nm)/TmPyPB (70 nm)/Liq (1 nm)/Al (100 nm). Here, dipyrazino [2,3-*f*:2′,3′-*h*]quinoxaline-2,3,6,7,10,11-hexacarbonitrile (HAT-CN) served as a hole injection layer and 1,1-bis((di-4-tolylamino)phenyl)-cyclohexane (TAPC) and *N*,*N*,*N*-tris(4-(9-carbazolyl)-phenyl)amine (TCTA) functioned as hole-transporting layers. 1,3,5-Tri(*m*-pyridin-3-ylphenyl) benzene (TmPyPB), 8-hydroxyquinolinolato-lithium (Liq), and aluminum (Al) were utilized as the electron-transporting layer, electron injection layer, and cathode, respectively. To facilitate triplet exciton recycling, a ternary system EML comprising a thermally activated delayed fluorescence (TADF) host, a phosphor sensitizer, and a terminal emitter was adopted. We employed 1,3-dihydro-1,1-dimethyl-3-(3-(4,6-diphenyl-1,3,5-triazin-2-yl)phenyl)indeno-[2,1-*b*]carbazole (DMIC-TRZ) as the TADF host.^39^ TADF materials as hosts, characterized by donor and acceptor moieties and a small Δ*E*_ST_, demonstrate the potential for achieving balanced charge injection and transporting mobilities concurrently, resulting in high efficiency with reduced efficiency roll-off.^40^ A red Os(II) complex, osmium(II) bis[3-(trifluoromethyl)-5-(4-*tert*-butylpyridyl)-1,2,4-triazolate] dimethylphenylphosphine [Os(bpftz)_2_(PPhMe_2_)_2_, OS1],^41,42^ was designed as a sensitizer to achieve significant spectral overlap and efficient energy transfer with the terminal emitters. The device structure and EL characteristics are depicted in Figures 6 and S27 and S28 of the Supporting Information, along with relevant parameters consolidated in Tables 1 and S3.

**Table 1:** Parameters of the Device Performance and EL Characteristics

emitter	*V*_on_^a^ (V)	λ_EL_^b^ (nm)	FWHM (nm/eV)^c^	EQE_max_^d^ (%)	CE_max_^e^ (cd/A)	PE_max_^f^ (lm/W)	CIE^g^ (*x*,*y*)
**DPNA**	3.8	666/725	40/0.11	12.23	2.37	1.92	0.70,0.30
**DPNA-F**	3.8	661/718	40/0.11	12.08	3.16	2.52	0.70,0.30
**DPNA-*^t^*Bu**	3.7	667/730	40/0.11	11.40	1.95	1.43	0.71,0.29

a *V*_on_ is the turn-on voltage measured at 0.1 cd/m^2^.

b λ_EL_ is the maximum EL peak.

c FWHM is the full width at half-maximum of electroluminescence.

d EQE_max_ is the maximum external quantum efficiency.

e CE_max_ is the maximum current efficiency.

f PE_max_ is the maximum power efficiency.

g CIEs are measured at 100 cd/m^2^. The outstanding high color purities of DPNAs are comparable to other reported red, narrowband OLEDs listed in Table S5. Especially, the FWHM in terms of energy (eV) is among the smallest ones.

The electroluminescent properties of the OS1 sensitizer were first investigated by fabricating the device without a terminal emitter (EML comprised DMIC-TRZ: 10 wt % OS1). EL performance is presented in Figure S27, exhibiting luminance (*L*) of 126,600 cd/m^2^ at 15.4 V (1105 mA/cm^2^), a peak maximum at 620 nm, and CIE coordinates of (0.65, 0.35). Additionally, maximum external quantum efficiency (EQE), current efficiency (CE), and power efficiency (PE) were recorded as 25.9%, 30.1 cd/A, and 28.3 lm/W, respectively. The OS1-based device maintained high EQEs of 22.5 and 20.1% at 1000 cd/m^2^ and 10,000 cd/m^2^, respectively, with small efficiency roll-off. This phenomenon stems from a wide charge recombination zone by the charge balance in EML and efficient energy transfer. As depicted in Figure S27c, a significant overlap between the emission of OS1 and the absorption of **DPNA** was observed.

To achieve deep-red and narrow emission, a low concentration of terminal emitters (**DPNA**, **DPNA-F**, and **DPNA-***^**t**^***Bu**) was incorporated into the EML. Figure 6 illustrates the sensitized devices with EML consisting of 10 wt % OS1 and 1 wt % terminal emitters in the DMIC-TRZ host. Sensitizer-free devices were also fabricated for comparison (Figure S28). However, these devices suffered from severe efficiency degradation (EQE_max_ of 2–3%) due to inefficient direct energy transfer from the host to the terminal emitter. The EL spectra displayed a weak shoulder peak at 460 nm originating from DMIC-TRZ. Upon doping with OS1 as a sensitizer, high EQE_max_ values of 12.2, 12.1, and 11.4% were observed for **DPNA**-, **DPNA-F**-, and **DPNA-***^**t**^***Bu**-based OLEDs, respectively. These OLEDs exhibited narrow emissions at 666, 661, and 667 nm with FWHM/CIE color coordinate values of 40 nm/(0.70, 0.30), 40 nm/(0.70, 0.30), and 40 nm/(0.71, 0.29), respectively, and ultrahigh brightness exceeding 10^4^ cd/m^2^. Note that in OLEDs, the film was prepared by co-deposition where **DPNAs** are only ca. 1–2% by weight. Therefore, one can treat this film as a **DPNAs** solid solution in the diluted condition, similar to that of **DPNAs** in solution. This, together with the lack of environmental perturbation for ESIPT, results in similar emission spectral features of **DPNAs** in both solution and film.

Moreover, all of the devices demonstrated strikingly stable EL spectra upon increased driving voltages (Figure S29). Notably, both the CIE coordinates closely approaching the pure-red BT.2020 gamut and the small FWHM compete with state-of-the-art MR emitters in devices, just as shown in Figure S31 and Table S5. Although intermolecular triplet to singlet transitions were spin-forbidden, the significant overlap between the EL spectra of OS1 with the absorption of **DPNA** (Figure S27c), as well as the small energy gap between the T_1_ of OS1 and the S_1_ of **DPNA**, enabled the breakthrough of the spin-forbidden transition from T_1_ to S_1_ via Förster resonance energy transfer (FRET) due to perturbation.^43,44^ However, an increased doping concentration led to a significant decrease in device efficiencies. When the doping concentration was increased to 2 wt %, EQE_max_ slightly decreased to 5–8%, attributed to unwanted Dexter energy transfer and aggregation-caused quenching (ACQ) of their planar structures. Furthermore, the horizontal dipole ratios (Θ_//_) for the EML films were measured (Figure S30), showing high values between 82 and 88%. This significant horizontal molecular orientation ratio (Θ_//_) can positively impact the optical outcoupling factor, thereby enhancing the EQEs of the devices.

## Conclusions

In summary, a comprehensive investigation of a series of double H-bonded red emitters, **DPNAs**, was carried out. Careful absorption and emission analyses provide unambiguous evidence of the ground-state isomerization among EE, EK, and KK isomers and the possible multiple intramolecular proton transfer in the excited state. Further, TGPL technique resolved the spectral temporal evolution of the ESIPT process within 100 fs, concluding that EK* → KK* ESIPT takes place with a rate of (157 fs)^−1^, and a stepwise EE* → EK* → KK* double proton transfer with an EE* → EK* rate of ∼(72 fs)^−1^, followed by the EK* → KK* rate of (157 fs)^−1^. The well-bound KK state with a rigid, large π-conjugated structure gives intense red emission. Moreover, the associated small reorganization energy enhances the 0–0 vibronic peak, narrowing the FWHM of the emission. In practical terms, using a sensitization method, the **DPNAs** associated OLEDs have resulted in superior performance, characterized by deep-red 660 nm emission with high EQEs, and more importantly, a narrow FWHM of 39–40 nm. The integration of fundamental studies with device engineering presents a promising strategy for ESIPT molecules in developing the color gamut in deep red and NIR in the future.

## Supporting Information Available

Experimental section and details of synthetic procedures and additional NMR spectra and crystal structure and other characterizations of DPNAs, computational approach, photophysical properties and experimental methods, and device fabrication and characterization (PDF)
