# Supplementary material for: Multiple Enol–Keto Isomerization and Excited-State Unidirectional Intramolecular Proton Transfer Generate Intense, Narrowband Red OLEDs
Source: J Am Chem Soc. 2024 Aug 23;146(35):24526–36. doi: 10.1021/jacs.4c07364 (PMC11378290; doi:10.1021/jacs.4c07364)
Supplement: Supplementary file 1 — ja4c07364_si_001.pdf [file ja4c07364_si_001.pdf]

Supporting Information

Multiple enol-keto isomerization and excited-state unidirectional intramolecular proton transfer generate intense, narrowband red OLEDs

Xiugang Wu*,+,1, Chih-Hsing Wang+,2, Songqian Ni+,1, Chi-Chi Wu+,2, Yan-Ding Lin[2], Hao-Ting Qu[2], Zong-Hsien Wu[3], Denghui Liu[4], Ming-Zhou Yang[1], Shi-Jian Su[4], Weiguo Zhu[1], Kai Chen[5], Zi-Cheng Jiang[6], Shang-Da Yang,*,6, Wen-Yi Hung*,3, Pi-Tai Chou*,2,7

Xiugang Wu, email: xgwu16@cczu.edu.cn

Shang-Da Yang, email: shangda@ee.nthu.edu.tw

Wen-Yi Hung, email: wenhung@mail.ntou.edu.tw

Pi-Tai Chou, email: chop@ntu.edu.tw

[1] School of Materials Science and Engineering, Jiangsu Engineering Laboratory of Light-Electricity-Heat Energy-Converting Materials and Applications, Changzhou University, Changzhou 213164, China.

[2] National Taiwan University, Department of Chemistry, Taipei, 10617, Taiwan.

[3] National Taiwan Ocean University, Department of Optoelectronics and Materials Technology, Keelung 20224, Taiwan.

[4] State Key Laboratory of Luminescent Materials and Devices and Institute of Polymer Optoelectronic Materials and Devices, South China University of Technology, Guangzhou, 510640, China.

[5] Robinson Research Institute, Faculty of Engineering, Victoria University of Wellington, Wellington, 6012, New Zealand

[6] National Tsing Hua University, Institute of Photonics Technologies, Hsinchu, 30013, Taiwan.

[7] Center for Emerging Material and Advanced Devices, National Taiwan University, Taipei, 10617, Taiwan.

+equal contribution

Table of Contents

1. **Experimental Section 0-1**
   General Information 0-1
   Reagents and materials 0-1
2. **Synthesis of the materials 0-2**
   Other characterizations 0-12
   Thermal properties 0-13
   Energy level characterization 0-13
3. **Photophysical Properties and Experimental Methods 0-16**
   Steady-State Spectra, PLQY, and Lifetime Measurements 0-16
   Transient grating photoluminescence (TGPL) 0-18
   Kinetic expression 0-19
4. **Computational Section 0-21**
5. **Device Fabrication and Characterization 0-26**
   OLED fabrication and Characterization 0-26
   Angle-Dependent PL Spectra Measurement and Simulation 0-26

1. Experimental Section

General Information

^{1}H and ^{13}C NMR spectra were measured on a Bruker Avance III 400 and 500 MHz NMR spectrometer using $CDCl_3$ as the solvent and tetramethylsilane as an internal standard at room temperature. A suitable crystal was selected, and three-dimensional X-ray data were collected on a Bruker D8 Venture diffractometer. The diffraction experiments were carried out at 100 K during data collection. Thermogravimetric analysis (TG-DTA) was performed by Bruker TG-DTA 2400SA with a heating rate of 10 °C min^{-1} from 40 °C to 600 °C under a nitrogen atmosphere. The UPS measurements were performed by PHI 5000 VersaProbe III with a base pressure of the analyzer chamber in the lower 10-8 Pa range. The UPS spectra were recorded using He-I radiation (photon energy EHe-I = 21.22 eV) generated in a differentially pumped, windowless discharge lamp. The measurements were performed with an applied bias of -9 V. To prepare the samples for UPS measurements, a 1 mg/mL solution of the small molecule in chlorobenzene was stirred at 50 °C for at least 1 h, in a nitrogen-filled glove box. ITO substrates were cleaned with isopropyl alcohol, acetone, detergent, and deionized water in an ultrasonic bath, followed by a 15-minute UV-ozone treatment, and the ITO substrate into 0.5 cm × 1 cm. The solution was subsequently spin-coated at 1000 rpm for 60 s inside the glove box on cleaned ITO substrates. Samples were transferred by air to the UPS setup.

Reagents and materials

Materials were purchased from commercial suppliers, and were used after appropriate purification, unless otherwise noted.

2. Synthesis of the materials

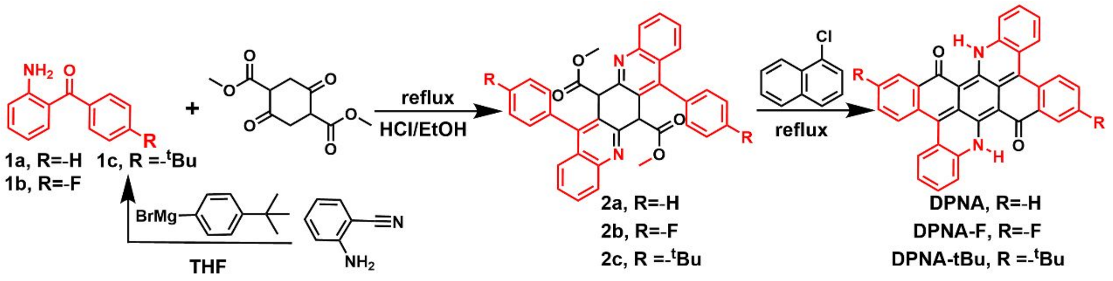

Figure S1. Synthetic route of DPNA, DPNA-F, and DPNA-^{t}Bu.

Synthesis of 1c: In a 500 mL double-necked flask, 4-tert-butylbromobenzene (12 g, 6.0 mmol), magnesium (1.61 g, 66 mmol), and iodine were added to 150 mL tetrahydrofuran (THF) and reacted at 78 °C under nitrogen protection for 2 hours. Then, a solution of 2-aminobenzonitrile (2 g, 16 mmol) in THF was slowly added to the reaction system at 0 °C, and the mixture was reacted for 0.5 hours. The reaction was then allowed to proceed at room temperature for 24 hours. After this, the mixture was extracted three times with dichloromethane. The combined organic layers were evaporated and dried, followed by column chromatography (eluent: petroleum ether/dichloromethane = 2/1), yielding 1.2 g of compound 1c with a yield of 30%. ^{1}H-NMR (400 MHz, $CDCl_3$) δ 7.63-7.58 (m, 2H), 7.52 -7.45 (m, 3H), 7.29 (ddd, J = 8.5, 7.1, 1.6 Hz, 1H), 6.74 (dd, J = 8.3, 1.2 Hz, 1H), 6.61 (ddd, J = 8.1, 7.0, 1.1 Hz, 1H), 6.03 (s, 2H), 1.36 (s, 9H). ^{13}C-NMR (101 MHz, $CDCl_3$) δ 198.92, 154.71, 150.76, 137.21, 134.57, 134.04, 129.28, 125.06, 118.49, 116.97, 115.50, 35.02, 31.24. MALDI-TOF MS (mass m/z): 253.789 $[M]^+$; calcd. for $C_{17}H_{19}NO$: 253.15.

Synthesis of 2a: 2-aminobenzophenone (19.7 g, 100 mmol), dimethyl 2,5-dioxocyclohexane-1,4-dicarboxylate (11.4 g, 50 mmol), 200 mL anhydrous ethanol and 4 mL hydrochloric acid were added to a 500 mL two-neck flask in turn, heated to 80 °C for reflux reaction for 12 hours under nitrogen atmosphere with stirring. After completing the reaction, the system was cooled to room temperature, methanol was added, solids were precipitated, vacuum filtering was carried out, and a large amount of methanol was used to wash the filter residue. After filtering, 23.29 g compound 2a was obtained with a yield of 84.7%. ^{1}H-NMR (400 MHz, $CDCl_3$) δ 8.16 (d, J =8.4 Hz, 2H), 7.71 (dp, J = 9.1, 4.8 Hz, 2H), 7.59-7.49 (m, 6H), 7.48-7.38 (m, 6H), 7.22 (dt, J = 7.8, 1.9 Hz, 2H), 5.42 (s, 2H), 3.62 (s, 6H). ^{13}C-NMR (101 MHz, $CDCl_3$) δ 170.09, 154.27, 148.57, 147.30, 135.45, 129.73, 129.55, 129.10, 129.04, 128.88, 128.81, 128.65, 127.34, 126.80, 126.70, 124.80, 54.03, 53.08. MALDI-TOF MS (mass m/z): 550.777 $[M]^+$; calcd. for $C_{36}H_{26}N_2O_4$: 550.19.

Synthesis of 2b: Similar to the synthesis of compound 2a, the procedure for compound 2b replaces 2-aminobenzophenone with 2-amino-4-fluorobenzophenone. The yield of the target compound 2b was 88.6%. ^{1}H-NMR (400 MHz, CDCl3) δ 8.17 (d, J = 8.5 Hz, 2H), 7.73 (ddd, J = 8.4, 6.6, 1.7 Hz, 2H), 7.50 – 7.40 (m, 6H), 7.24 (dtdd, J = 13.4, 8.2, 4.9, 2.0 Hz, 7H), 5.39 (s, 2H), 3.62 (s, 6H). 13C-NMR (101 MHz, CDCl3) δ 169.98 , 154.10 , 147.64 , 147.26 , 131.56 , 131.48 , 131.21 , 130.94 , 130.86 , 129.91 , 129.17 , 127.37 , 127.05 , 126.43 , 124.98 , 116.14 , 116.05 , 115.92 , 115.83 , 53.98 , 53.15 . MALDI-TOF MS (mass m/z): 586.517 $[M]^{+}$; calcd for C36H26F2N2O4: 586.17.

Synthesis of 2c: Similar to the synthesis of compound 2a, the procedure for compound 4 replaces 2-aminobenzophenone with compound 1c. The yield of the target compound 4 was 85.4%. ^{1}H-NMR (400 MHz, $CDCl_3$) δ 8.16 (d, *J* = 8.5 Hz, 2H), 7.70 (ddd, *J* = 8.4, 6.5, 1.6 Hz, 2H), 7.58 -7.35 (m, 10H), 7.18 -7.11 (m, 2H), 5.48 (s, 2H), 3.63 (s, 6H), 1.45 (s, 18H). ^{13}C-NMR (101 MHz, $CDCl_3$) δ 170.21, 154.44, 151.48, 132.27, 129.99-128.53 (m), 127.57, 126.79 (d, *J* = 26.2 Hz), 125.74 (d, *J* = 8.1 Hz), 54.08, 53.10, 34.84, 31.49. MALDI-TOF MS (mass m/z): 662.874 $[M]^{+}$; calcd for $C_{44}H_{42}N_2O_4$: 662.31.

Synthesis of DPNA: Compound 2a (5.5 g, 10 mmol) and 100 ml 1-chloronaphthalene were successfully added into a 250 mL two-nozzle flask, and the reaction was heated to 260 ℃ for 2 hours under nitrogen protection. After the reaction was complete, the system was cooled to room temperature, the solid was precipitated, the filter was extracted under reduced pressure, and the filter residue was washed with a large amount of methanol to obtain 4.28 g compound DPNA with a yield of 88%. MALDI-TOF MS (mass m/z): 486.147 $[M]^{+}$; calcd for $C_{34}H_{18}N_2O_2$: 486.14. Elemental analysis: C, 83.94; H, 3.73; N, 5.76; Found: C, 83.85; H, 3.76; N, 5.75.

Synthesis of DPNA-F: Similar to the compound DPNA, the difference is that compound 2a is replaced by compound 2b. The yield of the target compound DPNA-F was 83.2%. MALDI-TOF MS (mass m/z):522.137 $[M]^{+}$; calcd for $C_{34}H_{16}F_2N_2O_2$: 522.12. Elemental analysis: C, 78.16; H, 3.09; N, 5.36; Found: C, 78.26; H, 3.12; N, 5.35.

Synthesis of DPNA-tBu: Similar to the compound DPNA, the difference is that compound 2a is replaced by compound 2c. The yield of the target compound DPNA-tBu was 88.6%. MALDI-TOF MS (mass m/z): 598.431 [M]+; calcd for C42H34N2O2: 598.26. Elemental analysis: C, 84.25; H, 5.72; N, 4.68; Found: C, 84.26; H, 5.81; N, 4.67.

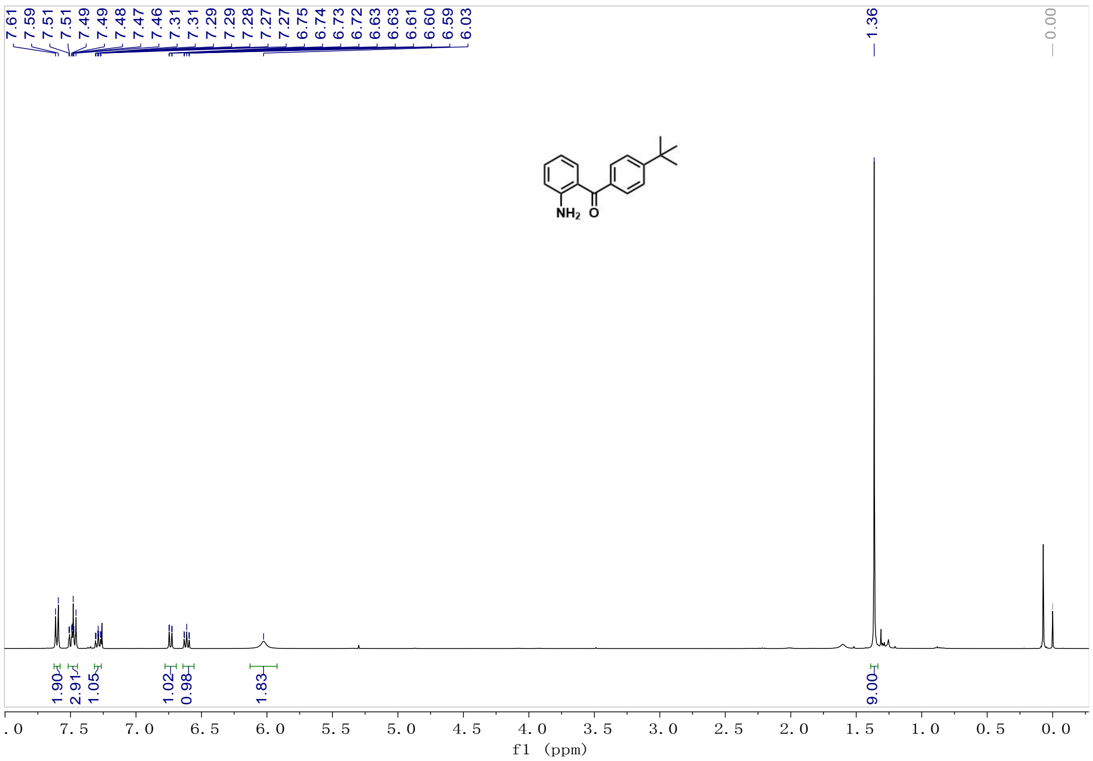

Figure S2. ^{1}H-NMR spectrum of compound 1c in $CDCl_3$ at room temperature.

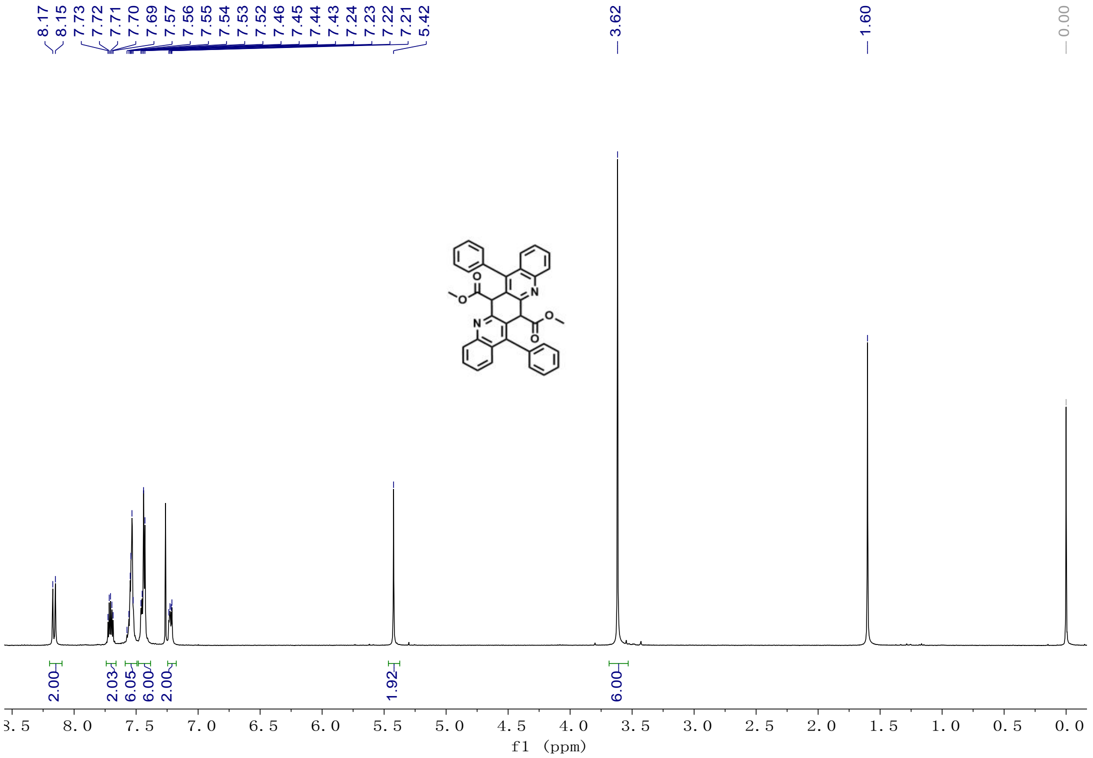

Figure S3. ^{1}H-NMR spectrum of compound 2a in $CDCl_3$ at room temperature.

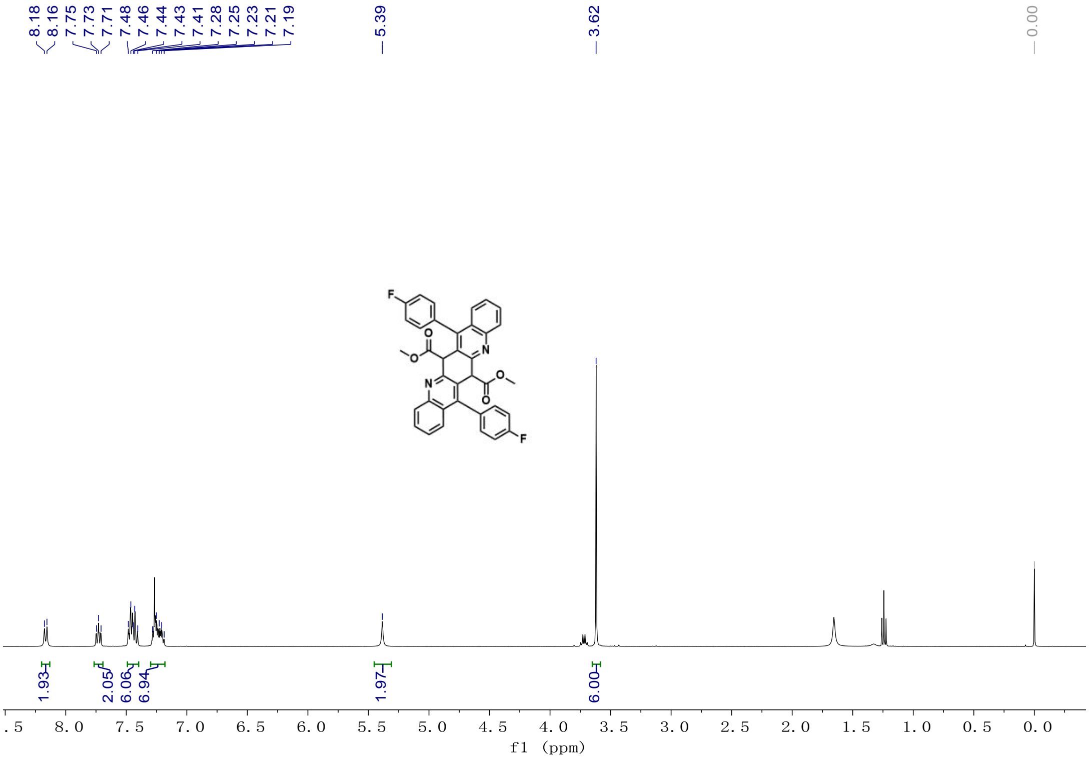

Figure S4. ^{1}H-NMR spectrum of compound 2b in $CDCl_3$ at room temperature.

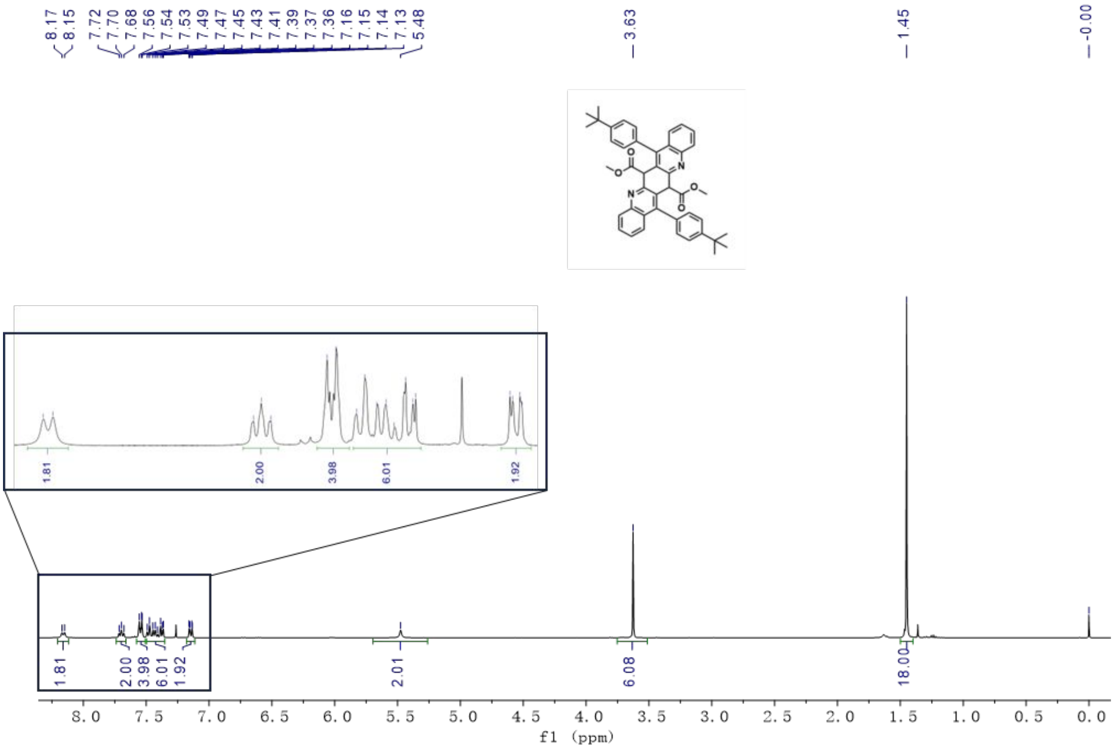

Figure S5. ^{1}H-NMR spectrum of compound 2c in $CDCl_3$ at room temperature.

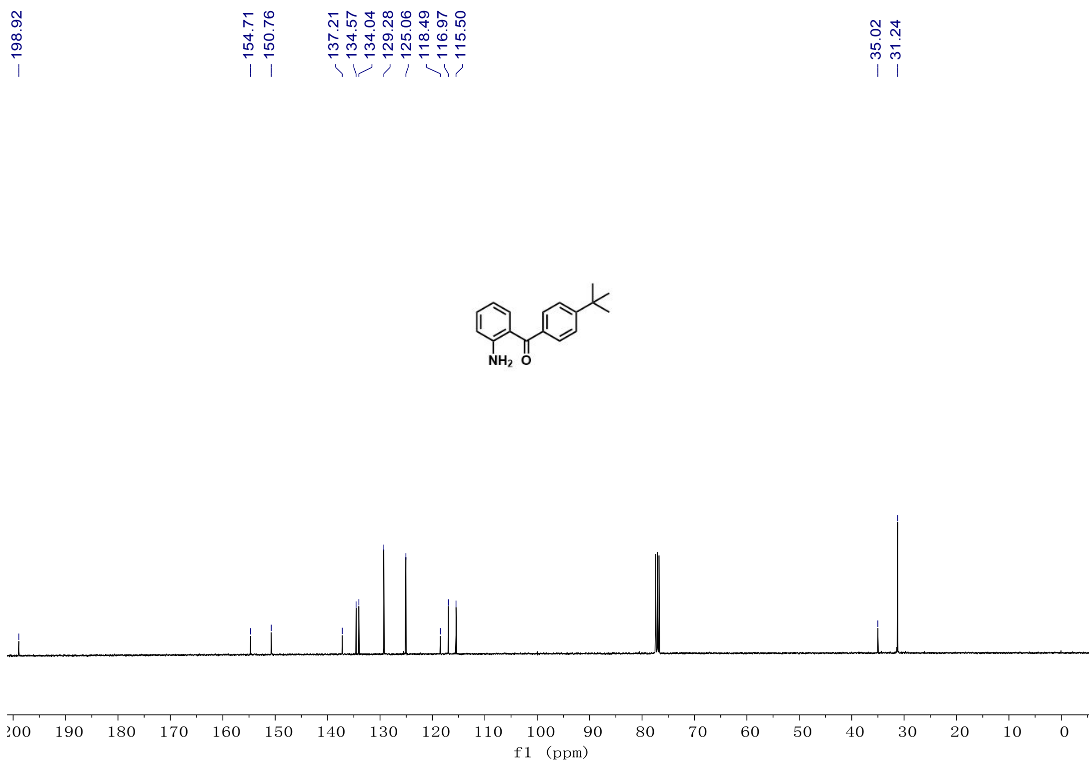

Figure S6. ^{13}C-NMR spectrum of compound 1a in $CDCl_3$ at room temperature.

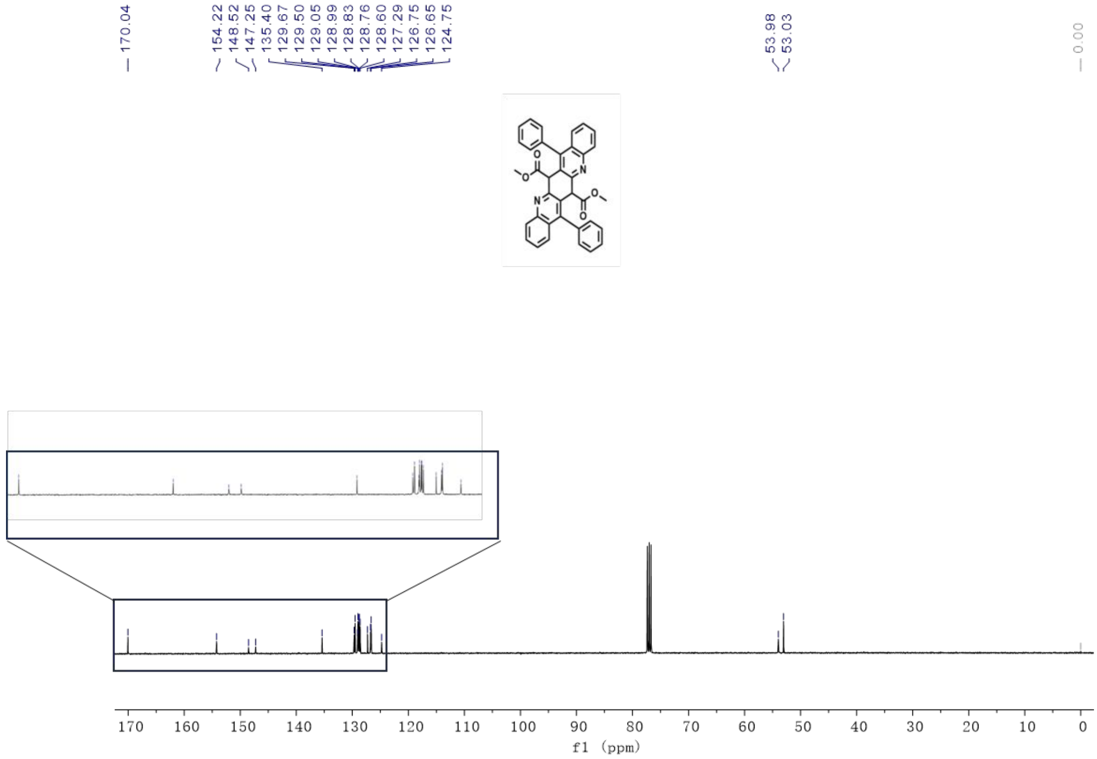

Figure S7. ^{13}C-NMR spectrum of compound 2a in $CDCl_3$ at room temperature.

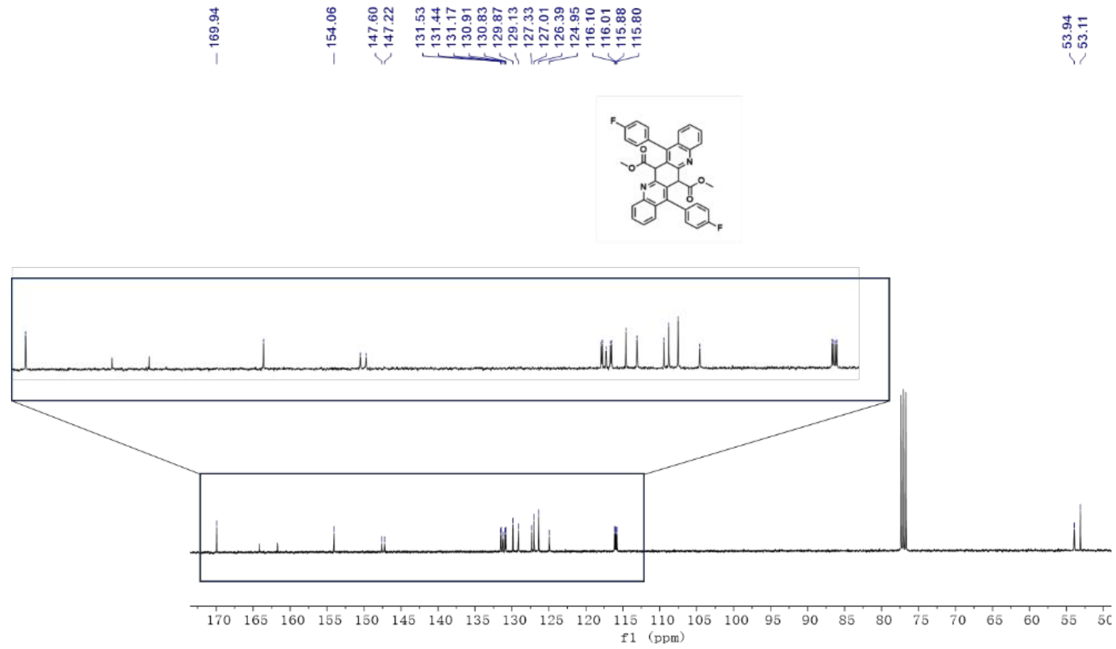

Figure S8. ^{13}C-NMR spectrum of compound 2b in $CDCl_3$ at room temperature.

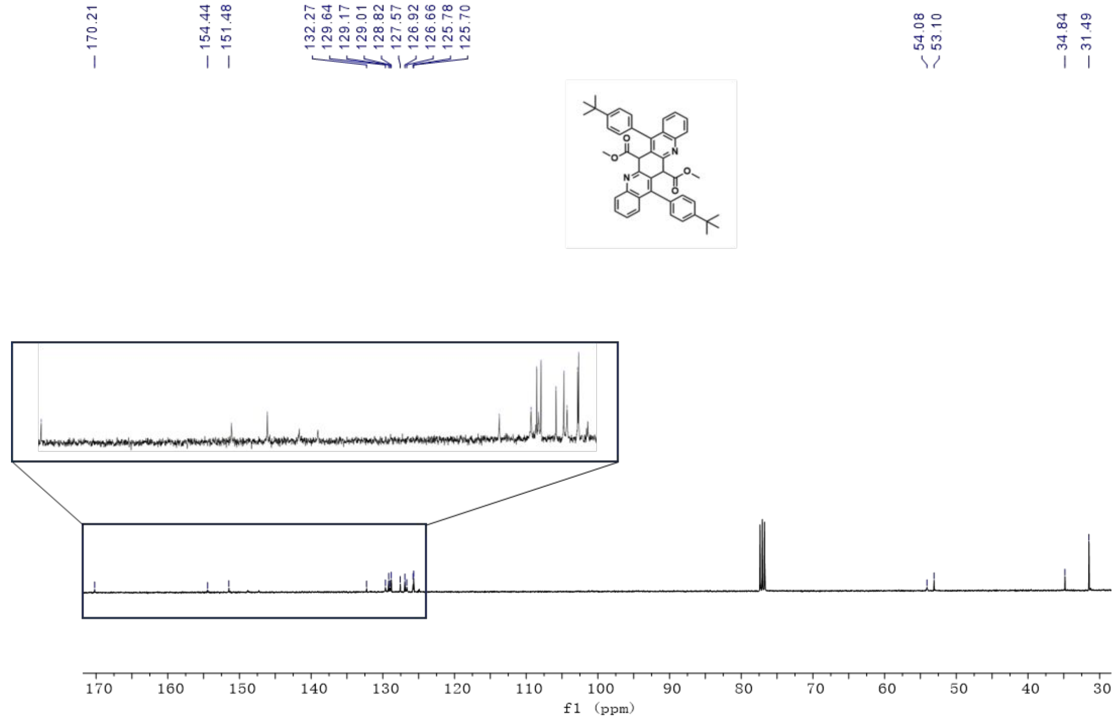

Figure S9. ^{13}C-NMR spectrum of compound 2c in $CDCl_3$ at room temperature.

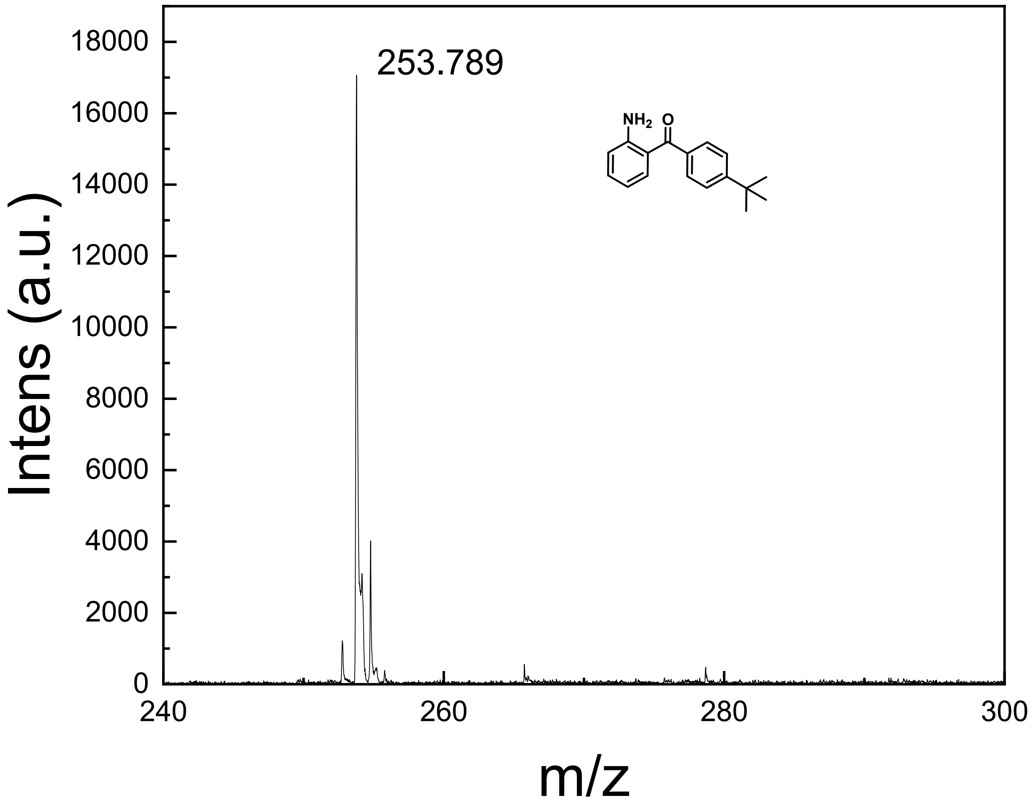

Figure S10. MS spectrum of compound 1c.

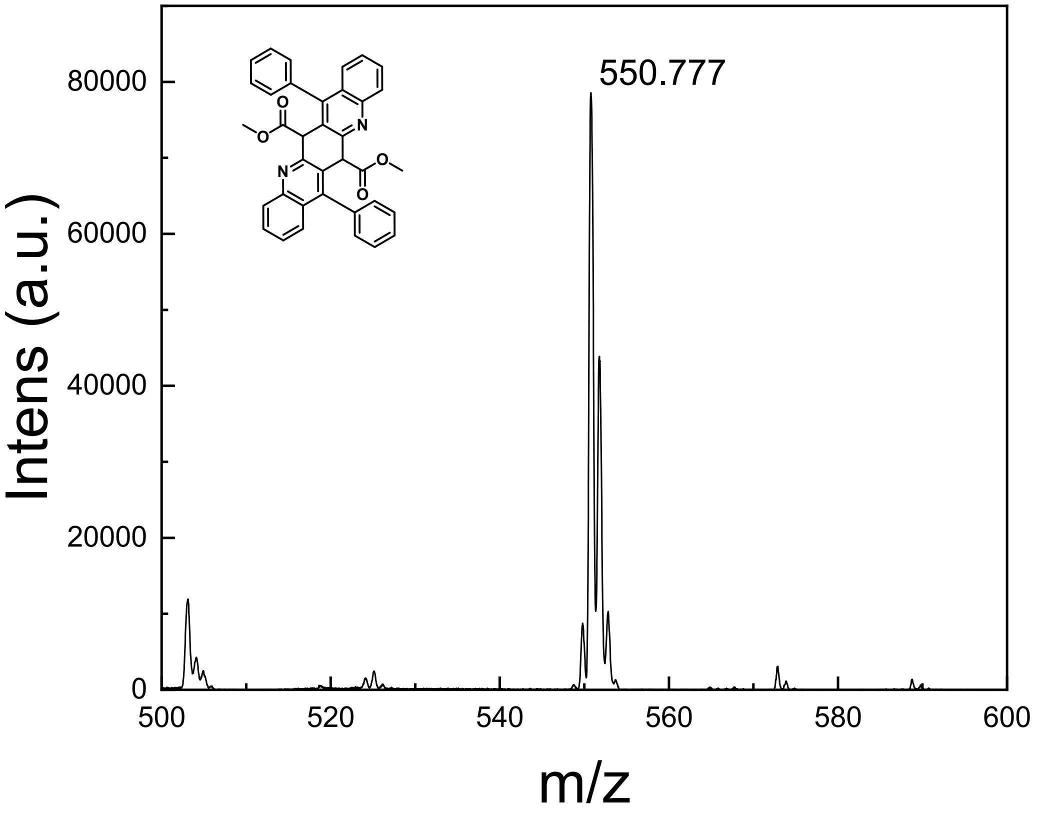

Figure S11. MS spectrum of compound 2a.

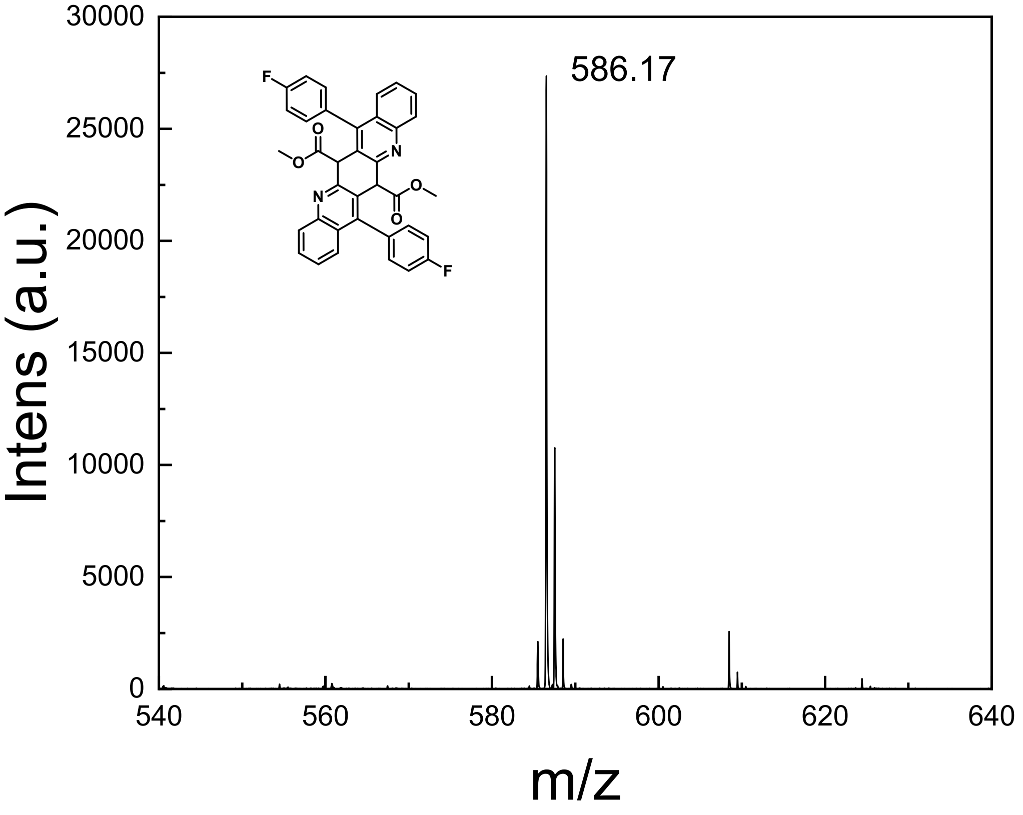

Figure S12. MS spectrum of compound 2b.

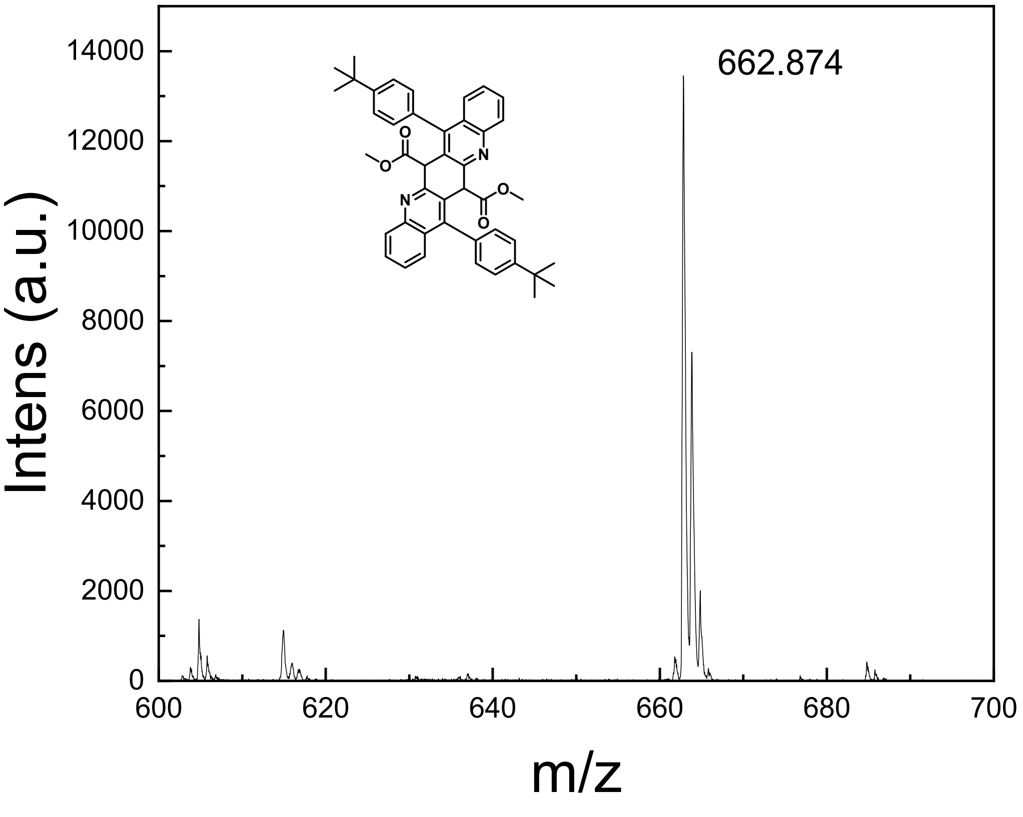

Figure S13. MS spectrum of compound 2c.

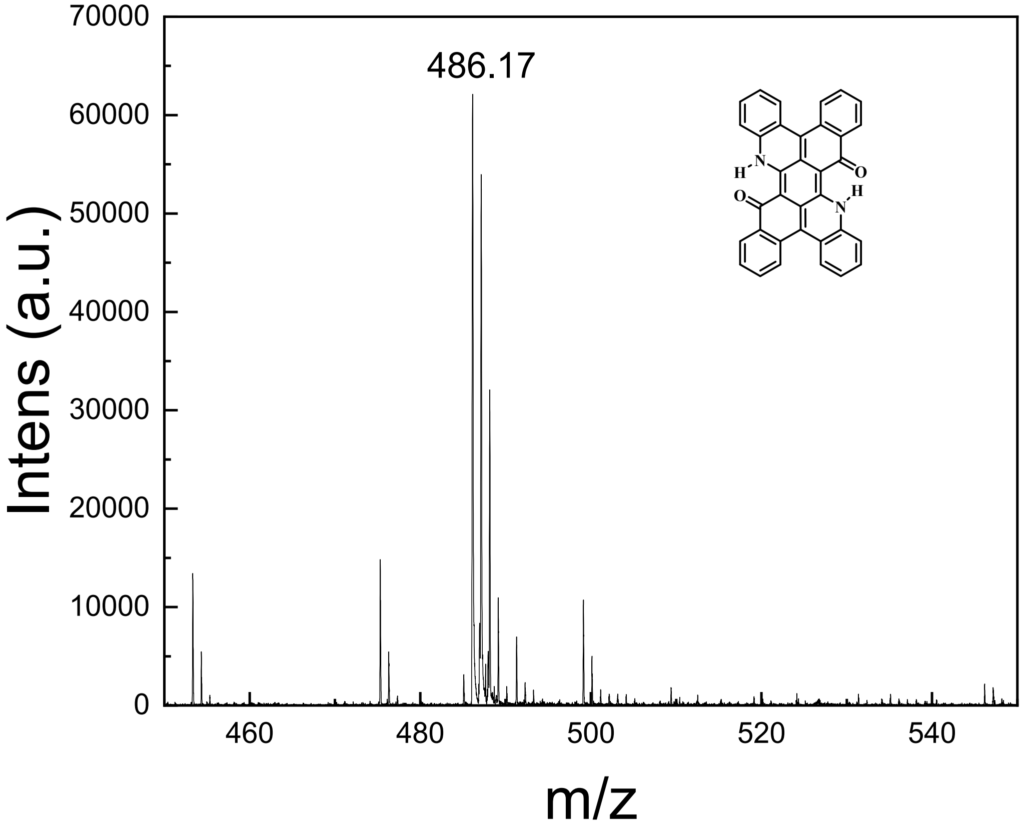

Figure S14. MS spectrum of DPNA.

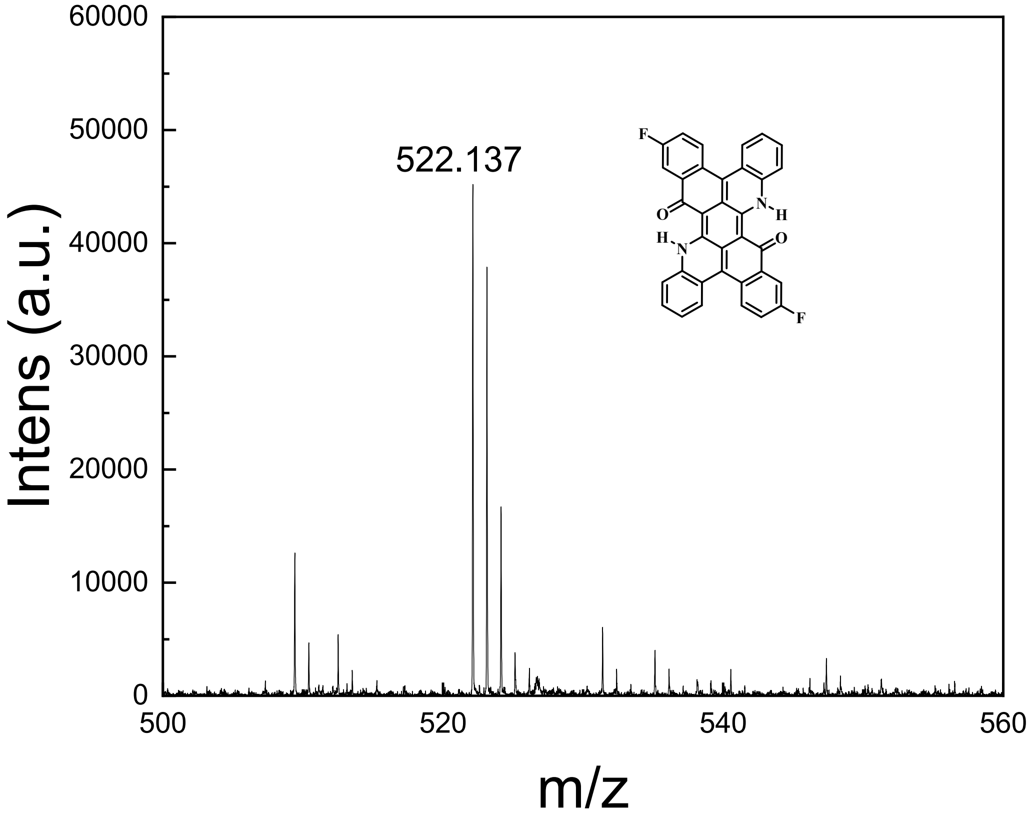

Figure S15. MS spectrum of DPNA-F.

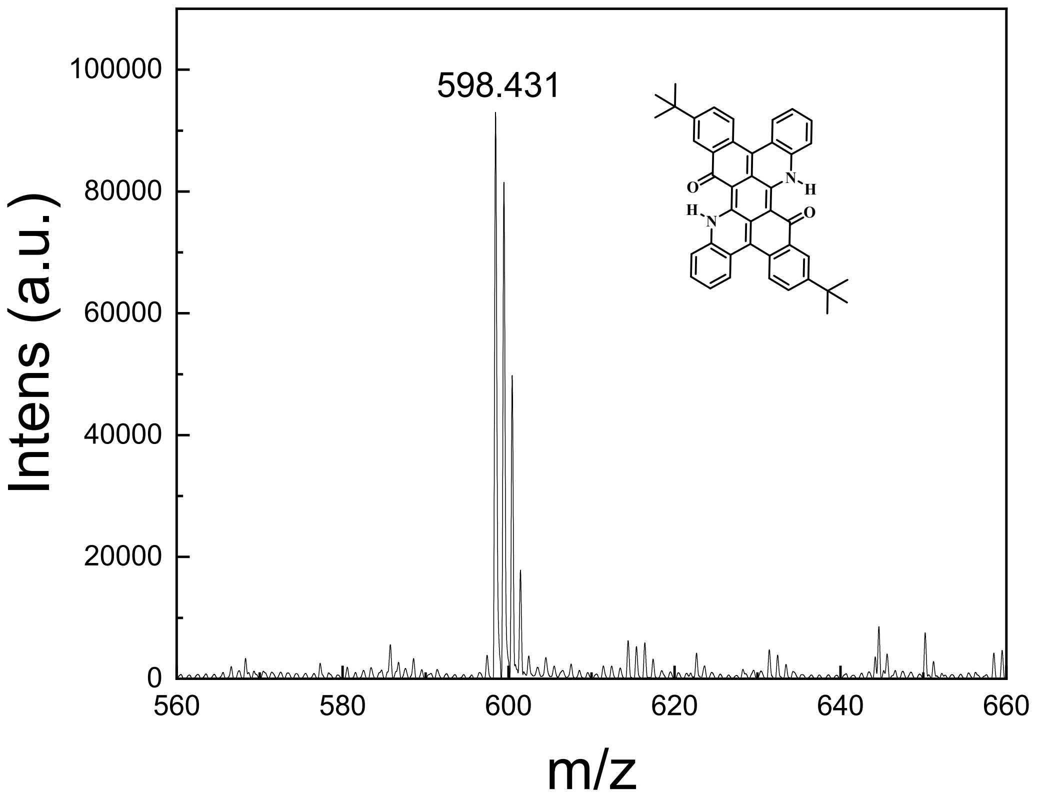

Figure S16. MS spectrum of DPNA-^{t}Bu.

Other characterizations

Table S1. Structure data of DPNA, DPNA-F, and DPNA-^{t}Bu single crystals.

Name	DPNA	DPNA-F	DPNA-^{t}Bu
Formula	$C_{34}H_{18}N_2O_2$	$C_{34}H_{16}F_2N_2O_2$	$C_{42}H_{34}N_2O_2$
Temperature/K	100	100	150
Crystal system	monoclinic	monoclinic	monoclinic
Space group	$P2_1/n$	$P2_1/c$	P-1
Cell Lengths (Å)	a= 10.5497 (5)<br>b=3.72810(10)<br>c=26.4220(11)	a= 3.69190 (10)<br>b=10.5107(2)<br>c=27.3938(6)	a= 7.2273(6)<br>b=11.5700(10)<br>c=18.1168(17)
Cell Angles (o)	α= 90<br>β= 95.087(2)<br>γ= 90	α= 90<br>β= 91.2120(10)<br>γ= 90	α= 82.104(5)<br>β=85.341(5)<br>γ=79.981(4)
Cell Volume (Å^3)	1035.09(7)	1062.76(4)	1475.1(2)
Z	2	2	2
Density (g/cm^3)	1.561	1.633	1.348
F(000)	504.0	536.0	632.0
Crystal size (mm^3)	0.12 × 0.08 × 0.05	0.12 × 0.08× 0.05	0.11 × 0.04 × 0.02
Radiation	MoKα (λ = 0.71073)	CuKα (λ = 1.54178)	CuKα (λ = 1.54178)
CCDC number	2355576	2355577	2355578

Thermal properties

General procedure for thermal measurements A TGA Q50 (TA instrument) thermal analysis system was employed to analyze thermal gravimetric analysis (TGA) ranging from 50 ℃ to 600 ℃ with a heating rate of 10°C min-1 under nitrogen flushing. The decomposition temperatures (T_d) of the emitters measured by TGA at 5% weight loss were observed at 466 ℃ (DPNA), 487℃ (DPNA-F), and 486 ℃ (DPNA-^{t}Bu).

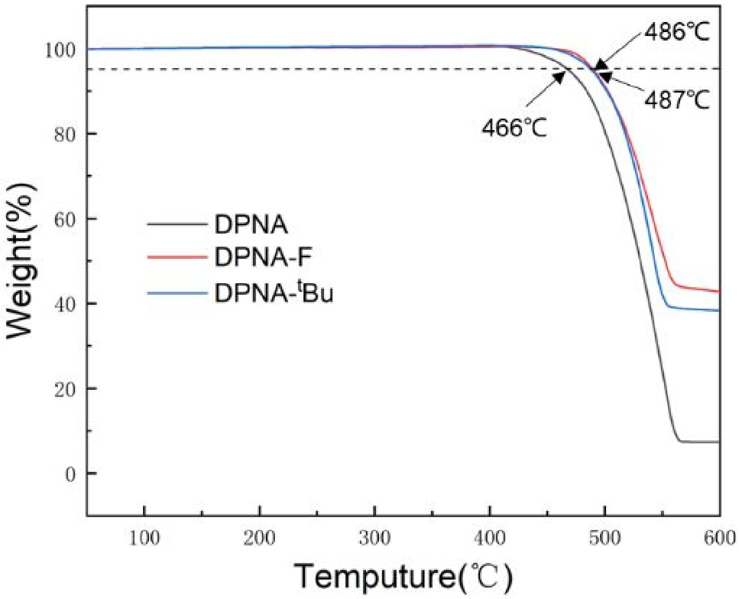

Figure S17. TGA thermograms of DPNA, DPNA-F, and DPNA-^{t}Bu at a heating rate of 10 °C min^{-1} under N_2.

Energy level characterization.

The UPS spectra were recorded using He-I radiation (photon energy EHe-I = 21.22 eV) generated in a differentially pumped, windowless discharge lamp. The measurements were performed with an applied bias of -9 V. To prepare the samples for UPS measurements, a 1 mg/mL solution of the small molecule in chlorobenzene was stirred at 50 °C for at least 1 h, in a nitrogen-filled glove box. ITO substrates were cleaned with isopropyl alcohol, acetone, detergent, and deionized water in an ultrasonic bath, followed by a 15-minute UV-ozone treatment, and the ITO substrate into 0.5 cm × 1 cm. The solution was subsequently spin-coated at 1000 rpm for 60 s inside the glove box on cleaned ITO substrates. Samples were transferred by air to the UPS setup.

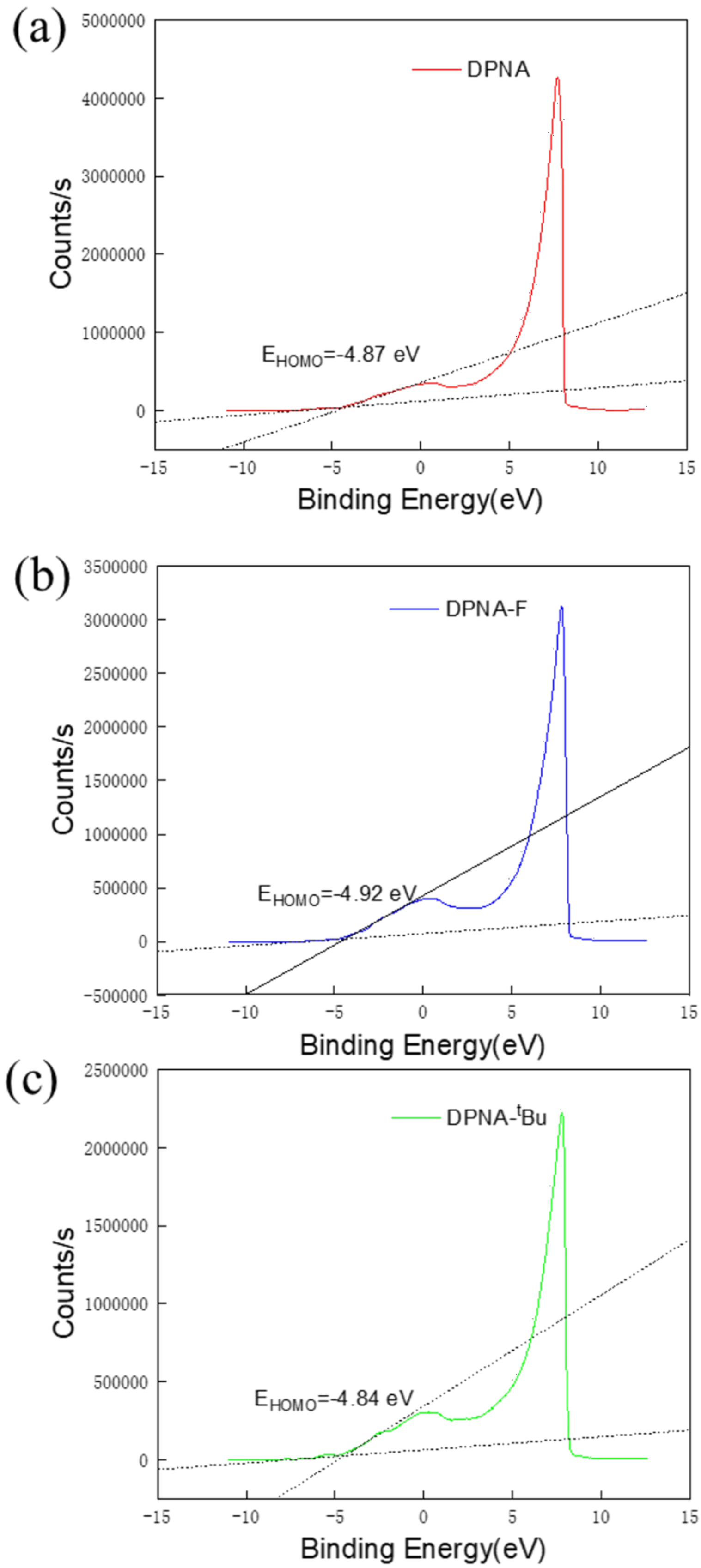

Figure S18. The UPS of (a) DPNA, (b) DPNA-F, and (c) DPNA-^{t}Bu were measured under the -9 V bias voltage.

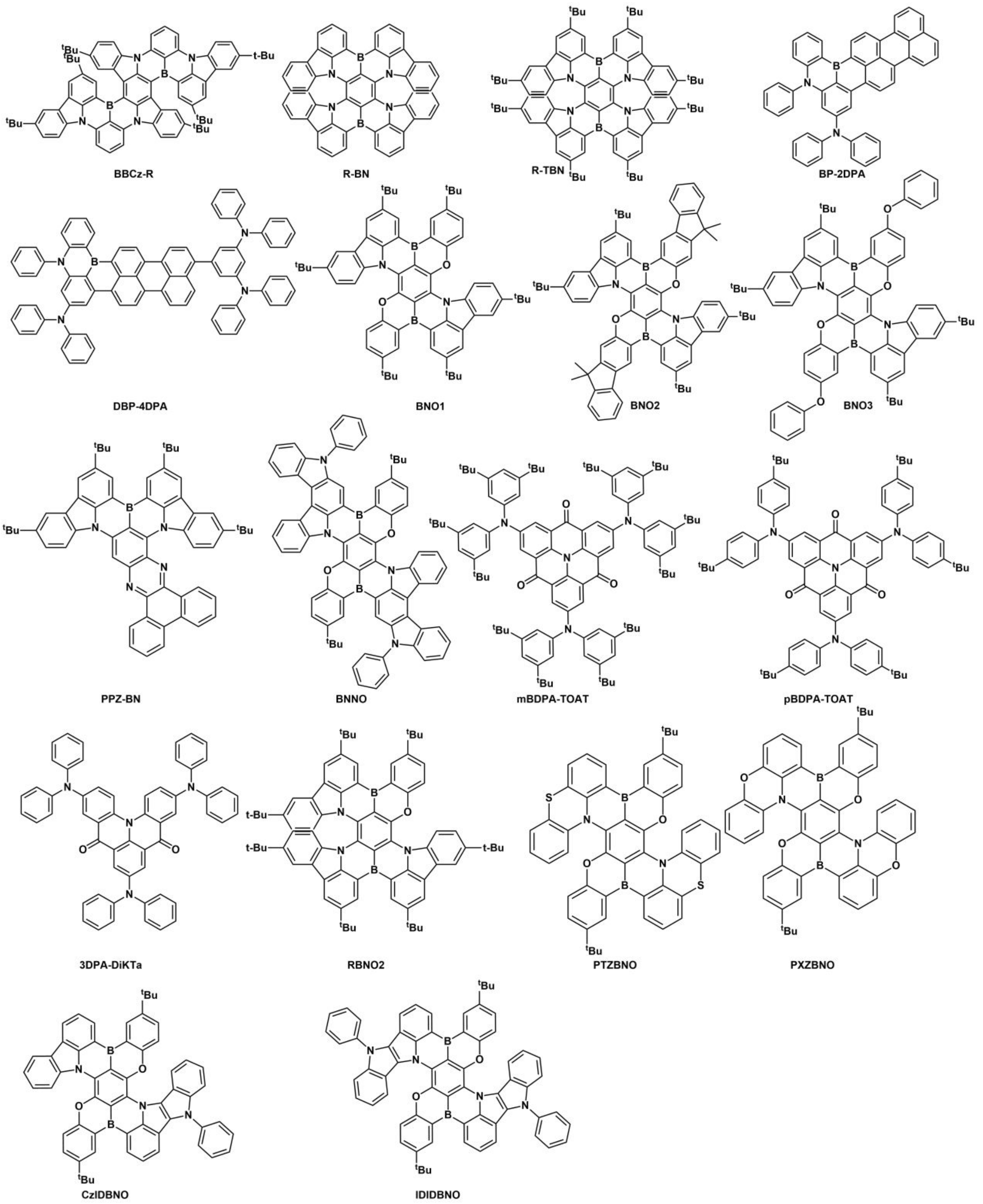

Figure S19. The molecular structure of representative red emitters with narrow bandwidth in OLEDs.

3. Photophysical Properties and Experimental Methods

Steady-State Spectra, PLQY, and Lifetime Measurements

Steady-state absorption and emission spectra were recorded by a double-beam spectrophotometer (Hitachi U-3310) and a fluorescence spectrometer (Edinburgh FS980), respectively. The photoluminescence quantum yields (PLQYs) were obtained by a comparative method relative to 4-(Dicyanomethylene)-2-methyl-6-(4-dimethylaminostyryl)-4*H*-pyran (Q.Y.=0.44) in methanol. The nanosecond time-resolved studies were performed by a time-correlated single photon counting (TCSPC) technique (Edinburgh FLS980) with a picosecond pulsed diode laser as the excitation light source. Both excitation and emission wavelengths of FLS980 were carefully calibrated. The samples were prepared in a 1-cm length cuvette with an absorbance of 0.1 (ca. 2×10^{-5} M) at the excitation wavelength.

The low-temperature spectra ranging from 77 to 298 K were recorded using a HITACHI UH5700 spectrometer, equipped with a custom-designed Temperature Controller 4000 Series 150 W (Specac) and a Four Port Variable Temperature Cuvette Holder (Specac). This configuration allows for precise sample placement and rigorous temperature control, maintaining a temperature accuracy within 2–3 K. At each temperature setting, reference spectra of the solvent were recorded to ensure accuracy. Methylcyclohexane-toluene in a 1:1 ratio was selected as the solvent due to its ability to form a transparent glass upon cooling, thereby facilitating clear spectral results.[1]

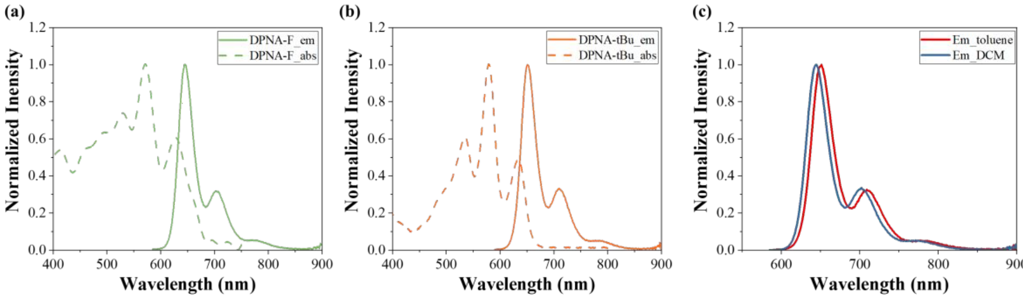

Figure S20. Absorption and emission spectra of (a) **DPNA-F** and (b) **DPNA-ᵗBu** in toluene (~ 1 × 10^{-5} M). (c) Emission spectra of DPNA in toluene and CH_2Cl_2. The excitation wavelength is 580 nm.

Table S2. Physical data of **DPNA**, **DPNA-F**, and **DPNA-ᵗBu**

Emitter	$\lambda_{abs}{}^{a}$ [nm]	$\lambda_{em}{}^{a}$ [nm]	ε^{d} [M^{-1} cm^{-1}]	FWHM^{a} [nm/eV]	Φ^{a} [%]	τ^{a} [ns]	k^{a} [$10^{8}s^{-1}$]	$k_{nr}{}^{a}$ [$10^{7}s^{-1}$]	HOMO^{b} [eV]	LUMO^{b} [eV]	$T_d{}^{c}$ [°C]
**DPNA**	535, 579, 635	651, 710, 780	1051	31/ 0.076	62.2	4.9	1.27	7.71	-4.87	-3.04	466
**DPNA-F**	530, 576, 633	650, 707, 773	1190	31/ 0.076	52.3	4.9	1.07	9.73	-4.92	-3.06	487
**DPNA-ᵗBu**	536, 580, 635	655, 715, 787	1983	32/ 0.076	72.8	4.6	1.58	5.91	-4.84	-3.03	486

Note: [a]Data were obtained in toluene. τ is acquired by excitation at 505 nm and monitoring at 650 nm. For more detail, see SI section 3; [b]Data for the HOMO levels were estimated using ultraviolet photoelectron spectroscopy (UPS), while the LUMO levels were calculated based on the optical energy gap; [c]T_d data were measured by TGA at 5% weight loss; [d]molar absorption coefficient (ε) at absorption peak wavelength in toluene.

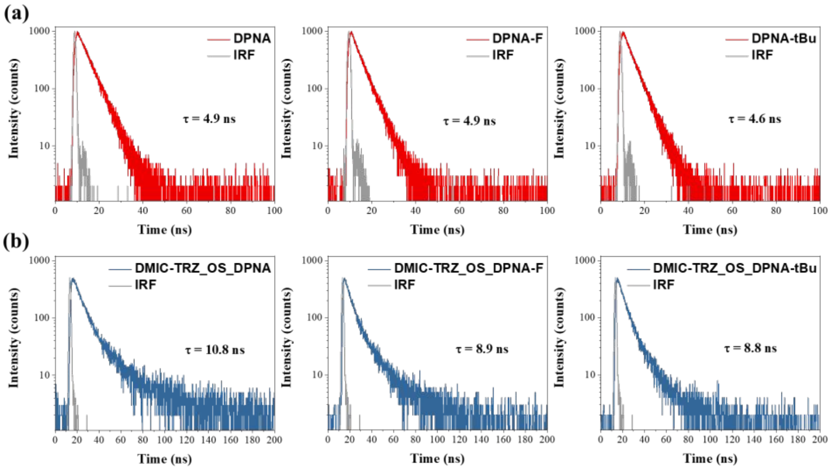

Figure S21. The decay lifetime of each sample was measured by TCSPC. (a) Data for DPNA, DPNA-F, and DPNA-ᵗBu toluene solution. The excitation wavelength is 505 nm, and the detector is monitored at 650 nm. (b) Data for DPNA, DPNA-F, and DPNA-ᵗBu 1% doped film. The excitation wavelength is 301 nm, and the detector is monitored at 650 nm.

Transient grating photoluminescence (TGPL)

Transient grating PL (TGPL) spectroscopy can achieve both high signal-to-noise ratio (SNR) and broad bandwidth simultaneously. In this scheme, a grating is formed by crossing two gate beams inside a $\chi^{(3)}$ crystal. This grating only lasts for a short time window comparable to the gate pulse width (≈50 fs in our system), within which all the colors of a broadband PL signal can be diffracted from the ungated PL beam. As a result, broad bandwidth and high SNR are achieved by the $\chi^{(3)}$ process (less constraint of phase mismatch) and angular separation between gated and un-gated PL signal, respectively

The output pulse from our home-built multiple-plate continuum (MPC) system with a pulse duration of 50 fs and wavelength of 1030 nm is separated into pump pulse and gate pulse using a pair of half-wave plate (HWP) and thin-film polarizer (TFP), which can also be used to gradually adjust the power splitting ratio by rotating the HWP.

For the gate beam path, the pulse is further divided into two pulses, whose height difference is adjusted by a roof mirror. After passing through a lens, these two gate beams are spatially overlapped onto the gate medium (fused silica with both sides polished) with a crossing angle.

For the pump beam path, the second-harmonic generation (SHG)/third-harmonic generation (THG) configuration can be switched according to the desired excitation wavelength. SHG (515 nm excitation) is generated by a 150-µm-thick BBO crystal, with a maximum pulse energy of up to 100 nJ. In the case of THG, a dual waveplate is inserted after the SHG module, making fundamental (1030 nm) and second harmonic (515 nm) beams linearly polarized along the same direction. A group delay compensation plate compensates for the temporal delay between the 1030 nm and 515 nm pulses. Finally, sum-frequency generation occurs in a type I BBO crystal, leading to third-harmonic pulses at 343 nm. A bandpass filter can be used to eliminate 515 nm and 1030 nm light. The polarization and pulse energy of the pump beam can be adjusted by a pair of HWP and wire-grid polarizers.

After generating the excitation pulse, a lens is employed to focus the beam onto the sample. Subsequently, the fluorescence light is collected and refocused onto the gate medium using two off-axis parabolic mirrors, respectively. The fluorescence from the sample and two gate beams form a conventional boxcar geometry for spatially filtering the ungated signals and gate beams, allowing only the gated signal to enter the polychromator equipped with a spectrometer (SP2300, Acton) and intensified CCD (ICCD, PI-MAX 3, Princeton Instruments).

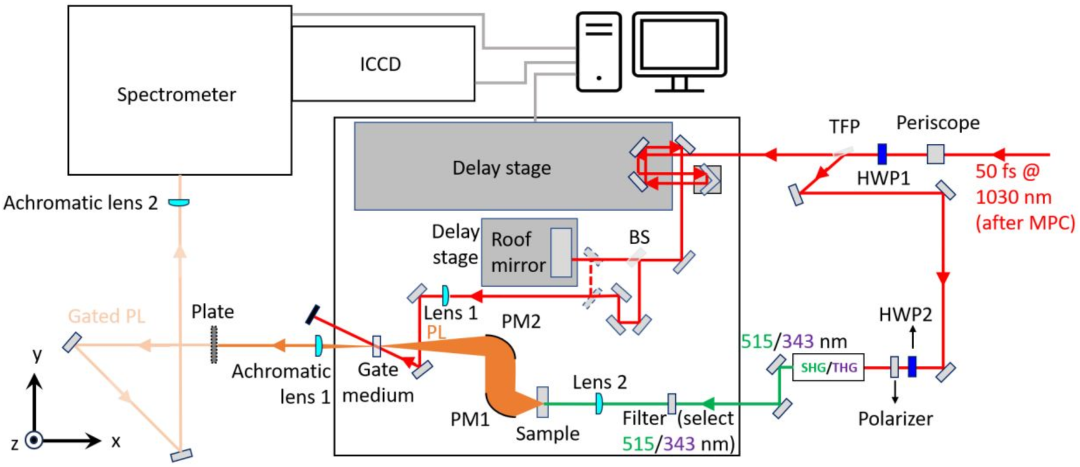

Figure S22. TGPL setup: MPC, multiple-plate continuum; HWP#, half-wave plate; TFP, thin-film polarizer; SHG, second-harmonic generation; THG, third-harmonic generation; PM#, parabolic mirrors; BS, beam splitter; PL, photoluminescence, ICCD, intensified charge-coupled device

Kinetic expression

"The fitting methodology is based on the kinetic expression for $EE^* \rightarrow EK^* \rightarrow KK^*$ shown in Figure 5 in the text. Based on the proposed mechanism (see Figure 5), ESIPT is irreversible. Thus, the corresponding reaction kinetics can be depicted below.

$$\frac{d[EE^*]}{dt} = -k_{EE}[EE^*] - k_{PT1}[EE^*]$$

$$\frac{d[EK^*]}{dt} = k_{PT1}[EE^*] - k_{EK}[EK^*] - k_{PT2}[EK^*]$$

$$\frac{d[KK^*]}{dt} = k_{PT2}[EK^*] - k_{KK}[KK^*]$$

where $[EE^*]$, $[EK^*]$, and $[KK^*]$ are concentrations of each isomer in the S_1 state. k_{PT1} and k_{PT2} are the rate constants of proton transfer, while k_{EE}, k_{EK}, and k_{KK} are the observed decay rate constants of each form. The kinetics process can be further simplified by assuming that the proton transfer rates of EE* and EK* are faster than the emission rate, i.e., k_{PT1}, $k_{PT2} >> k_{EE}$, k_{EK}, k_{KK}. This $EE^* \rightarrow EK^* \rightarrow KK^*$ is considered to be a stepwise process. Therefore, the reaction kinetics can be written as:

$$\frac{d[EE^*]}{dt} = -k_{PT1}[EE^*]$$

$$\frac{d[EK^*]}{dt} = k_{PT1}[EE^*] - k_{PT2}[EK^*]$$

$$\frac{d[KK^*]}{dt} = k_{PT2}[EK^*]$$

By solving these differential equations, we can determine the concentrations of EE*, EK*, and KK* over time, which describe the kinetics of the stepwise ESIPT reaction. The above differential equation can be solved by direct differentiation, and the solution is shown as follows.

$$[EE^*](t) = [EE^*](0)\ e^{-k_{PT1}t}$$

$$[EK^*](t) = \frac{k_{PT1}}{k_{PT2} - k_{PT1}}[EE^*](0)\ (e^{-k_{PT1}t} - e^{-k_{PT2}t})$$

$$[KK^*](t) = [EE^*](0)\left(1 - \frac{k_{PT1}}{k_{PT2}-k_{PT1}}\ (e^{-k_{PT1}t} - e^{-k_{PT2}t}) - e^{-k_{PT1}t}\right)$$ “

4. Computational Section

Table S3. The computed optical excitations and molecular orbital contributions for EE, EK and KK forms of all title compounds.

		no.	E/eV	nm	f	Contribution	weight
DPNA	EE @s0-opt	S_1	2.56	483.5	0.5168	HOMO→LUMO	96.6%
		S_2	2.80	442.5	0.0013	HOMO-1→LUMO	11.2%
						HOMO→LUMO+1	86.6%
		T_1	1.88	659.9	0	HOMO→LUMO	86.0%
		T_2	2.18	568.6	0	HOMO→LUMO+1	87.0%
	EK @s0-opt	S_1	2.34	530.1	0.5485	HOMO→LUMO	97.9%
		S_2	2.63	471.1	0.021	HOMO→LUMO+1	97.1%
		T_1	1.60	775.2	0	HOMO→LUMO	97.0%
		T_2	2.00	619.8	0	HOMO-1→LUMO+1	7.0%
						HOMO→LUMO+1	85.0%
	KK @s0-opt	S_1	2.12	585.6	0.5950	HOMO→LUMO	99.2%
		S_2	2.58	480.4	0	HOMO→LUMO+1	96.2%
		T_1	1.27	999.8	0	HOMO→LUMO	100.0%
		T_2	2.01	616.2	0	HOMO→LUMO+1	94.0%
DPNA-F	EE @s0-opt	S_1	2.57	482.6	0.5069	HOMO→LUMO	96.5%
		S_2	2.79	444.3	0.0012	HOMO-1→LUMO	11.8%
						HOMO→LUMO+1	86.2%
		T_1	1.87	663.9	0	HOMO→LUMO	86.0%
		T_2	2.17	570.8	0	HOMO-1→LUMO+2	5.0%
						HOMO→LUMO+1	86.0%
	EK @s0-opt	S_1	2.39	526.0	0.5368	HOMO→LUMO	97.9%
		S_2	2.60	475.9	0.0216	HOMO→LUMO+1	97.1%
		T_1	1.61	767.9	0	HOMO→LUMO	97.0%
		T_2	1.98	626.2	0	HOMO-1→LUMO+1	8.0%
						HOMO→LUMO+1	84.0%
	KK @s0-opt	S_1	2.13	582.0	0.586	HOMO→LUMO	99.2%
		S_2	2.54	487.6	0	HOMO→LUMO+1	96.4%
		T_1	1.29	961.2	0	HOMO→LUMO	100.0%
		T_2	1.97	628.8	0	HOMO→LUMO+1	94.0%
DPNA-^{t}Bu	EE @s0-opt	S_1	2.54	488.3	0.5701	HOMO→LUMO	96.8%
		S_2	2.77	447.3	0.0013	HOMO-1→LUMO	8.2%
						HOMO→LUMO+1	89.6%
		T_1	1.88	659.5	0	HOMO→LUMO	88.0%
		T_2	2.15	577.3	0	HOMO-1→LUMO+2	6.0%
						HOMO→LUMO+1	88.0%
	EK	S_1	2.33	531.4	0.6076	HOMO→LUMO	97.9%
		S_2	2.61	475.3	0.0217	HOMO→LUMO+1	97.2%

@s0-opt	T_1	1.61	771.8	0	HOMO→LUMO	97.0%
	T_2	1.98	626.6	0	HOMO-1→LUMO+1	6.0%
					HOMO→LUMO+1	85.0%
KK @s0-opt	S_1	2.11	587.8	0.6598	HOMO→LUMO	99.2%
	S_2	2.55	486.0	0	HOMO→LUMO+1	96.4%
	T_1	1.28	968.1	0	HOMO→LUMO	100.0%
	T_2	1.98	625.5	0	HOMO→LUMO+1	95.0%

		HOMO	LUMO	LUMO+1
DPNA	EE @s0-opt	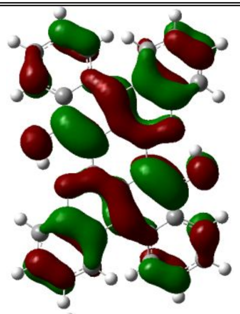	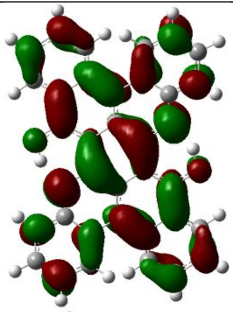	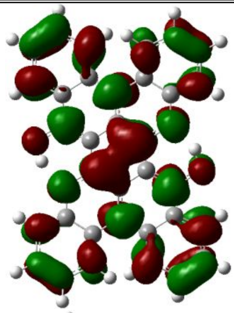
	EK @s0-opt	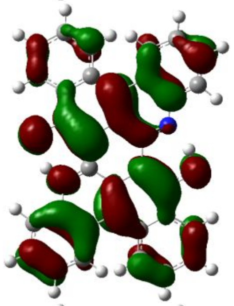	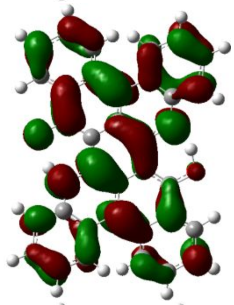	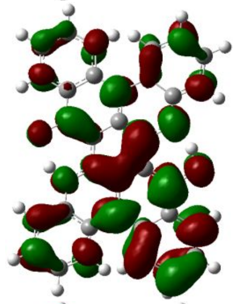
	KK @s0-opt	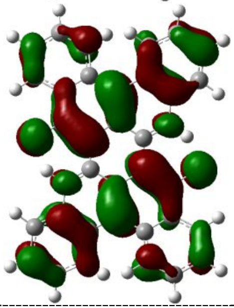	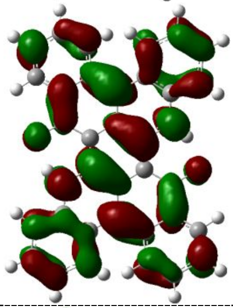	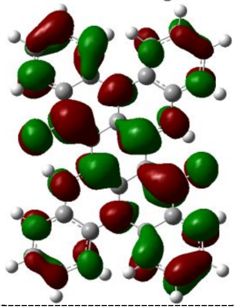
DPNA-F	EE @s0-opt	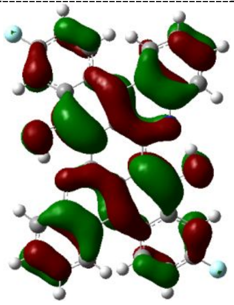	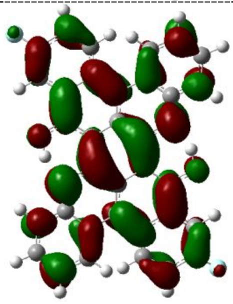	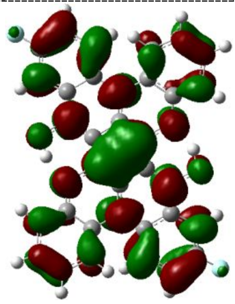

EK @s0-opt

KK @s0-opt

DPNA-^{t}Bu

EE @s0-opt

EK @s0-opt

KK @s0-opt

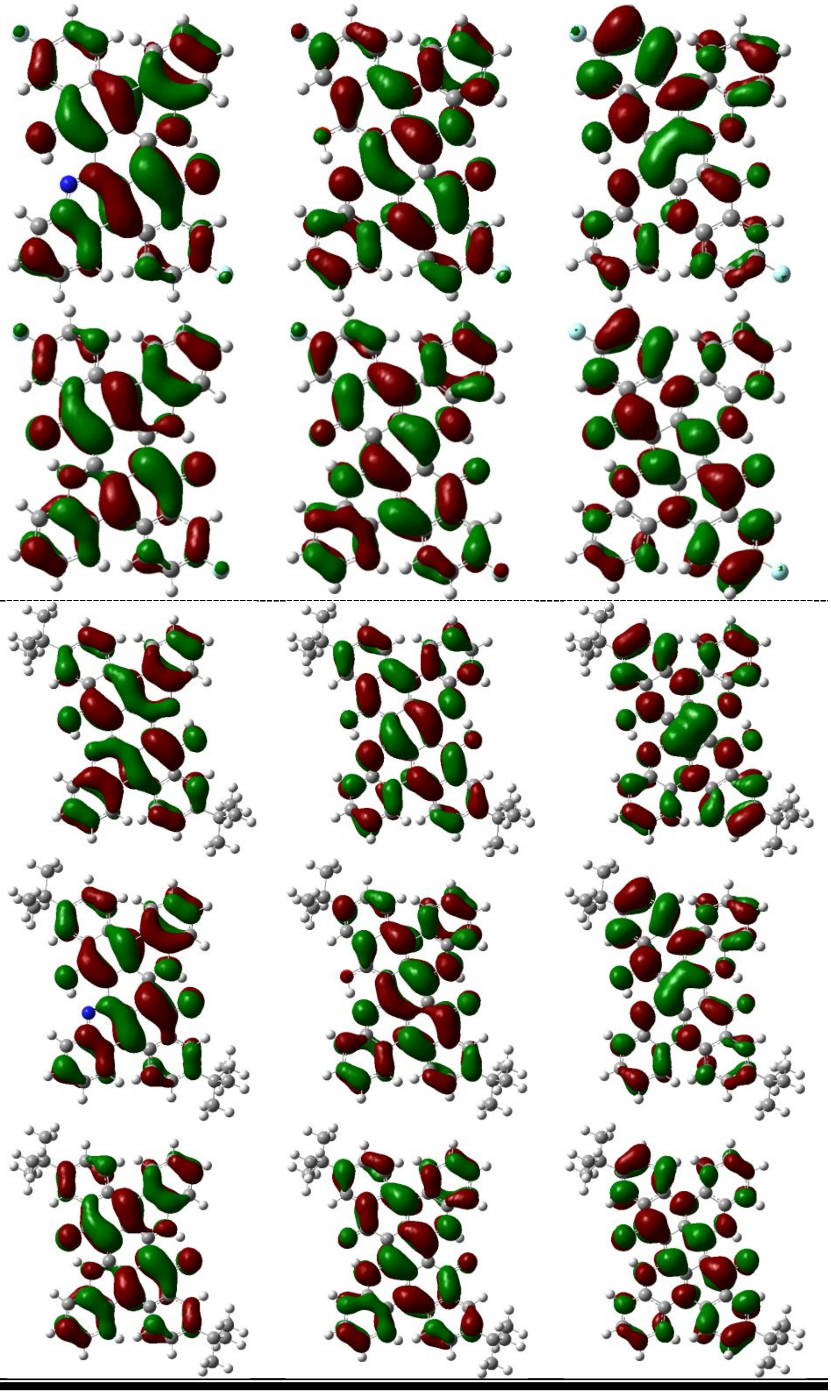

Figure S23. Frontier molecular orbitals associated with major optical transitions of EE, EK and KK forms at S_0-optimized structure for all title compounds.

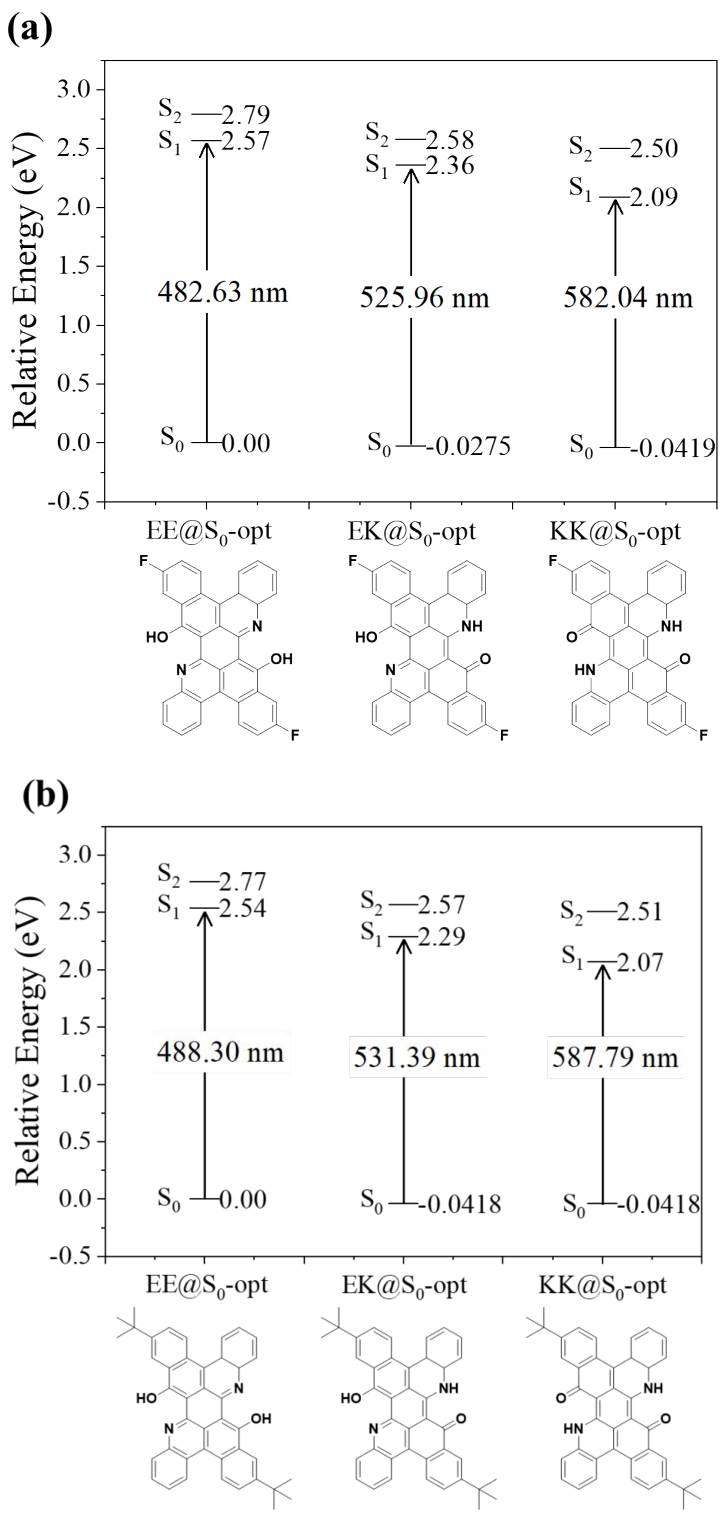

Figure S24. Energy Diagram of EE, EK and KK forms at S_0-optimized, noted as "@S0-opt", of (a) **DPNA-F** and (b) **DPNA-^{t}Bu.**

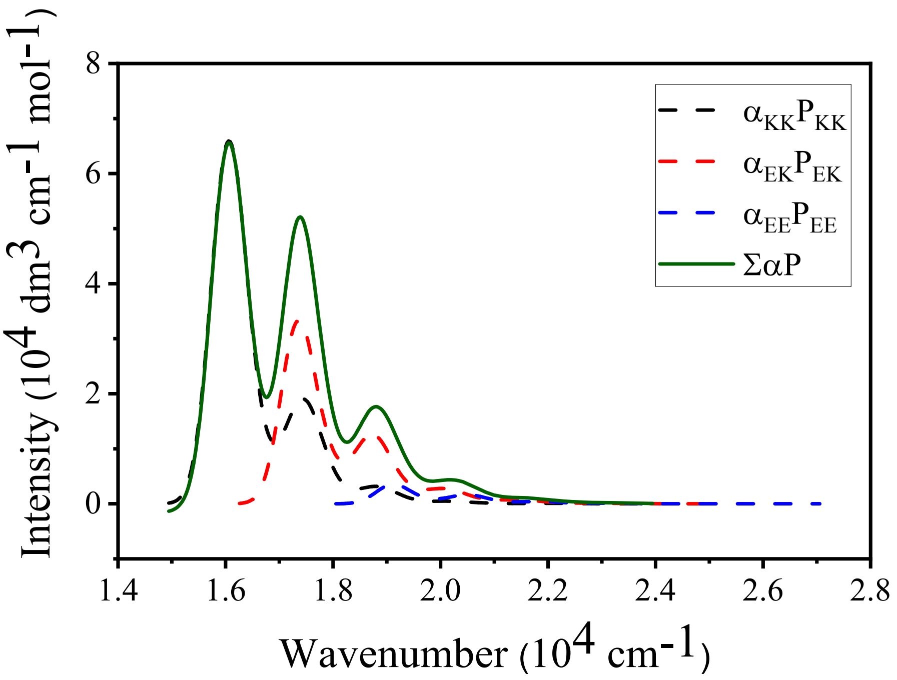

Figure S25. The simulated absorption spectra of **DPNA**.

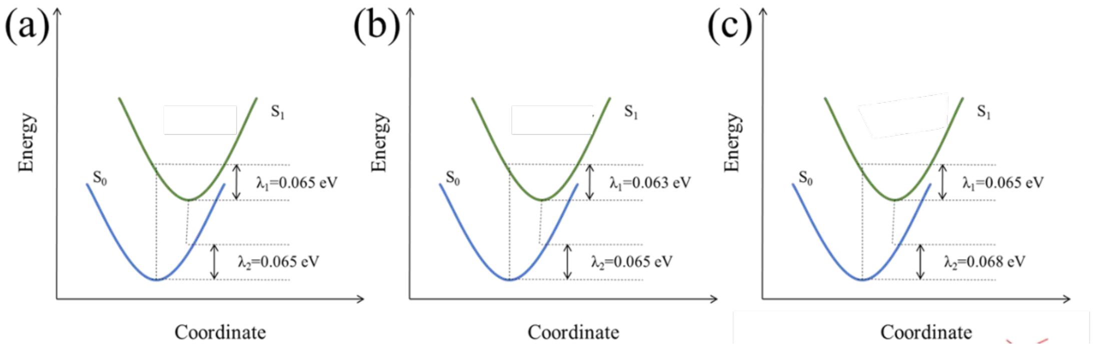

Figure S26. The calculated internal reorganization energy (λ) of keto-keto (KK) isomer of (a) **DPNA**, (b) **DPNA-F**, and (c) **DPNA-^{t}Bu** based on the optimized structures conducted by DFT and TD-DFT under the B3LYP/DEF2SVP level for the S_0 and S_1states, respectively.

The reorganization energies were calculated by Nelsen's four-point method. The corresponding equation is $\lambda = \lambda_1+\lambda_2$, where $\lambda_1$and λ_2 denotes the molecular geometry relaxation energy of vertical transition from the excited state to the ground state (and vice versa). [2]

5. Device Fabrication and Characterization

OLED fabrication and Characterization

All compounds were purified by vacuum sublimation before fabrication. The OLEDs were created through vacuum deposition of materials at 10^{-6} torr onto ITO-coated glass substrates with a sheet resistance of 15 Ω sq^{-1}. The ITO surface was ultrasonically cleaned with acetone, methanol, and deionized water in sequence, and finally with N_2 plasma. The deposition rate for each organic material was approximately 1–2 Å·s^{-1}. The J–V–L characteristics of the devices were measured in a glove box. The device's driving source was a programmable source measurement unit (Keithley 2614B), and light intensity was measured using a calibrated silicon detector. Each EL spectrum was collected using an optical fiber connected to a spectrometer (Instrument Systems CAS 125).

Angle-Dependent PL Spectra Measurement and Simulation

The angular-dependent *p*-polarized PL intensity was measured to analyze the transition dipole moment of the EML. The experimental setup included a motorized rotation stage, a fused silica half-cylindrical lens, a longpass filter to block the excitation beam, a polarizer, and a fiber-guided spectrometer (Instrument Systems CAS 125) to collect the polarized emission. A continuous-wave Nd: YAG laser (355 nm, 10 kHz) served as the excitation source, fixed at an incident angle of 45°. The sample was deposited on a quartz substrate and encapsulated under an N_2 atmosphere. Measurements were performed automatically using the programmed rotation stage, recording the *p*-polarized emission intensity from 0° to 90° in 5° increments. The data were analyzed with SETFOS 4.5 (Fluxim AG, Switzerland) to determine the ratio of horizontal dipoles (Θ).

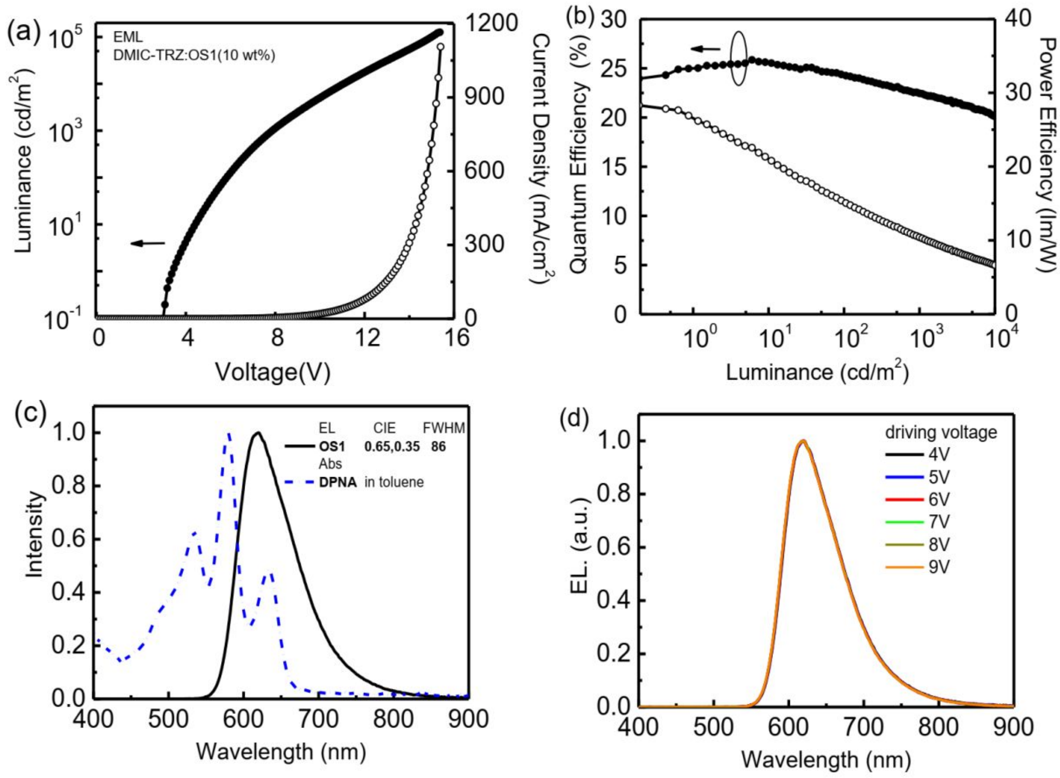

Figure S27. (a) J-V-L characteristics, (b) EQE–PE–L characteristics, (c) the spectral overlap between emission (OS1) and absorbance (**DPNA** in Tol), and (d) EL spectra at various voltages. Device structure: ITO/ HATCN (5nm)/ TAPC (30nm)/ TCTA (10nm)/ DMIC-TRz: OS1(10 wt%) (20nm)/ TmPyPB (70nm)/ Liq/ Al.

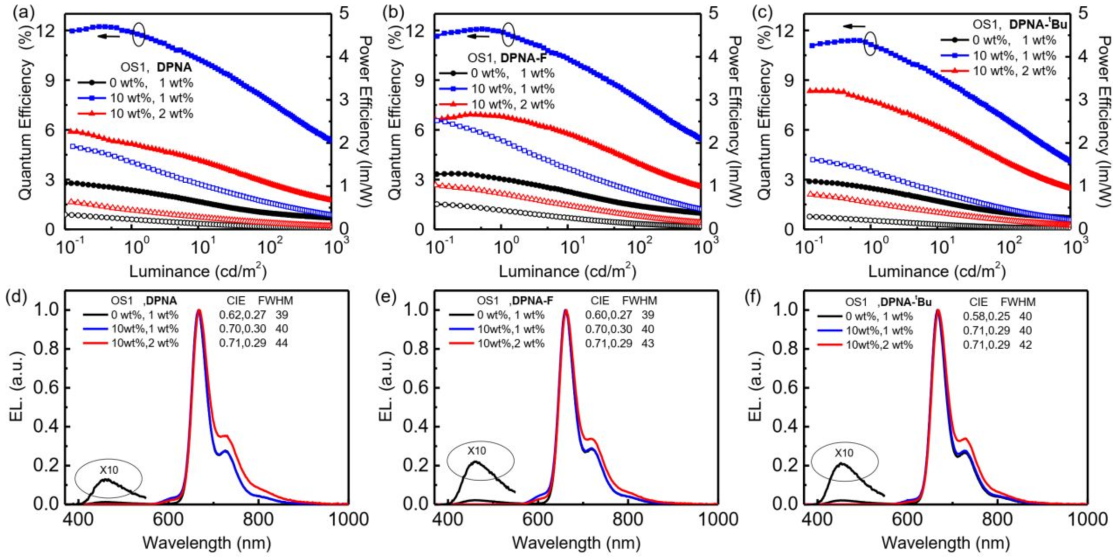

Figure S28. (a-c) EQE–PE–L characteristics, and (d-f) normalized EL spectra of devices fabricated using **DPNA**, **DPNA-F**, and **DPNA-^{t}Bu** as the terminal emitter.

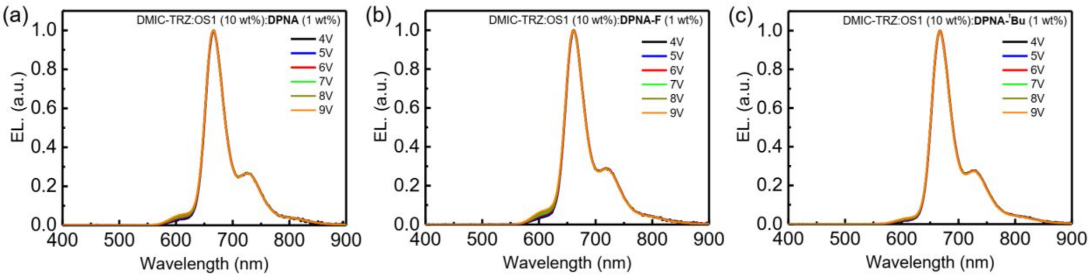

Figure S29. EL spectra of devices fabricated using 1 wt% (a) **DPNA**, (b) **DPNA-F,** and (c) **DPNA-^{t}Bu** as terminal emitters at various driving voltages.

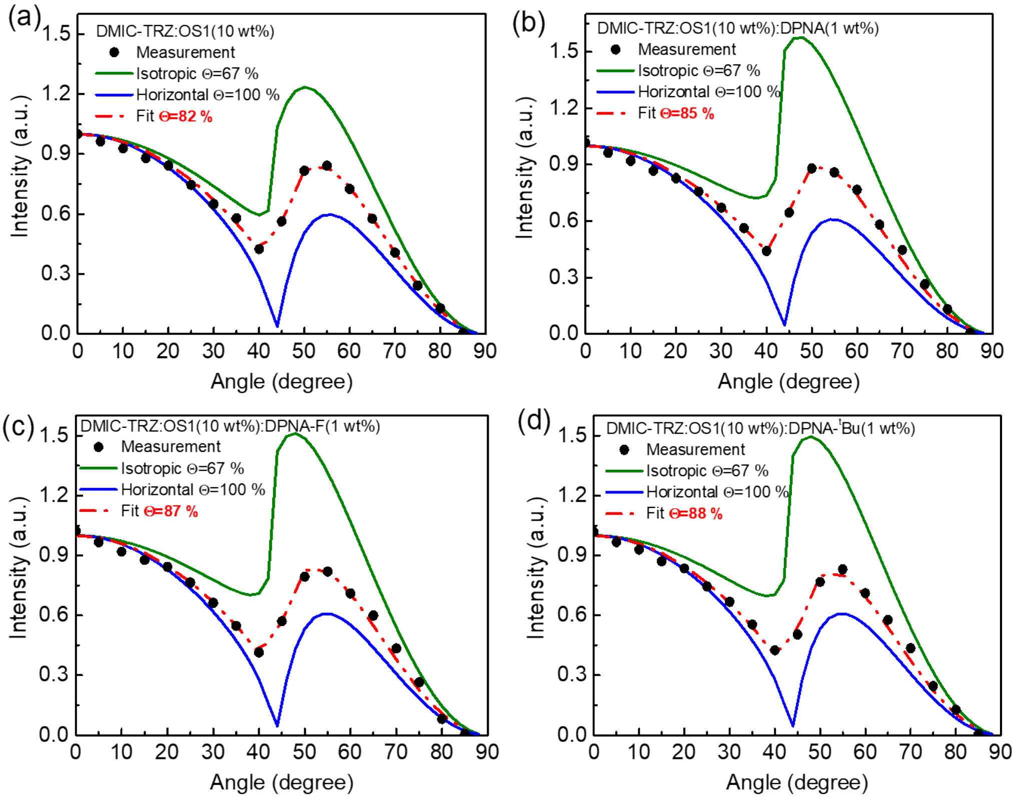

Figure S30. The characterizations of horizontal dipole orientation by angle-dependent *p*-polarized PL spectra. Measured horizontal transition dipole moment ratios of (a) 10 wt% OS1, (b) 10wt% OS1: 1wt% **DPNA**, (c) 10wt% OS1: 1wt% **DPNA-F** and (d) 10wt% OS1: 1wt% **DPNA-^{t}Bu** doped in DMIC-TRZ deposited film.

Table S4. The overall performance of EL Performances of the device.

Weight Os1	Terminal emitter		V_{on} [V]	EQE/CE/PE max [%/cd A^{-1}/lm W^{-1}]	EQE/CE/PE/V at 10^3 cd m^{-2} [%/cd A^{-1}/lm W^{-1}/V]	λ_{max} [nm]	FWHM [nm]	CIE [x,y]
10 wt%	none		3.1	25.9/30.1/28.3	22.5/26.2/10.4/7.9	620	86	0.65,0.35
none	**DPNA**	1 wt%	4.3	2.79/0.46/0.34	0.70/0.12/0.03/13.5	460, 666,726	39	0.62,0.27
10 wt%		1 wt%	3.8	12.23/2.37/1.92	5.37/1.04/0.27/12.3	666,725	40	0.70,0.30
10 wt%		2 wt%	4.2	5.89/0.84/0.63	1.77/0.25/0.06/14.4	667,725	44	0.71,0.29
none	**DPNA -F**	1 wt%	4.2	3.36/0.76/0.58	0.99/0.22/0.05/13.3	460, 661,718	39	0.60,0.27
10 wt%		1 wt%	3.8	12.08/3.16/2.52	5.47/1.43/0.38/11.9	661,718	40	0.70,0.30
10 wt%		2 wt%	4.1	6.93/1.39/1.02	2.57/0.52/0.12/13.6	663,717	43	0.71,0.29
none	**DPNA -^{t}Bu**	1 wt%	4.4	2.89/0.41/0.29	1.77/0.25/0.06/14.4	460, 667,730	40	0.58,0.25
10 wt%		1 wt%	3.7	11.40/1.95/1.43	4.12/0.71/0.17/13.2	667,730	40	0.71,0.29
10 wt%		2 wt%	4.3	8.35/1.13/0.81	2.48/0.34/0.07/15.8	668,730	42	0.71,0.29

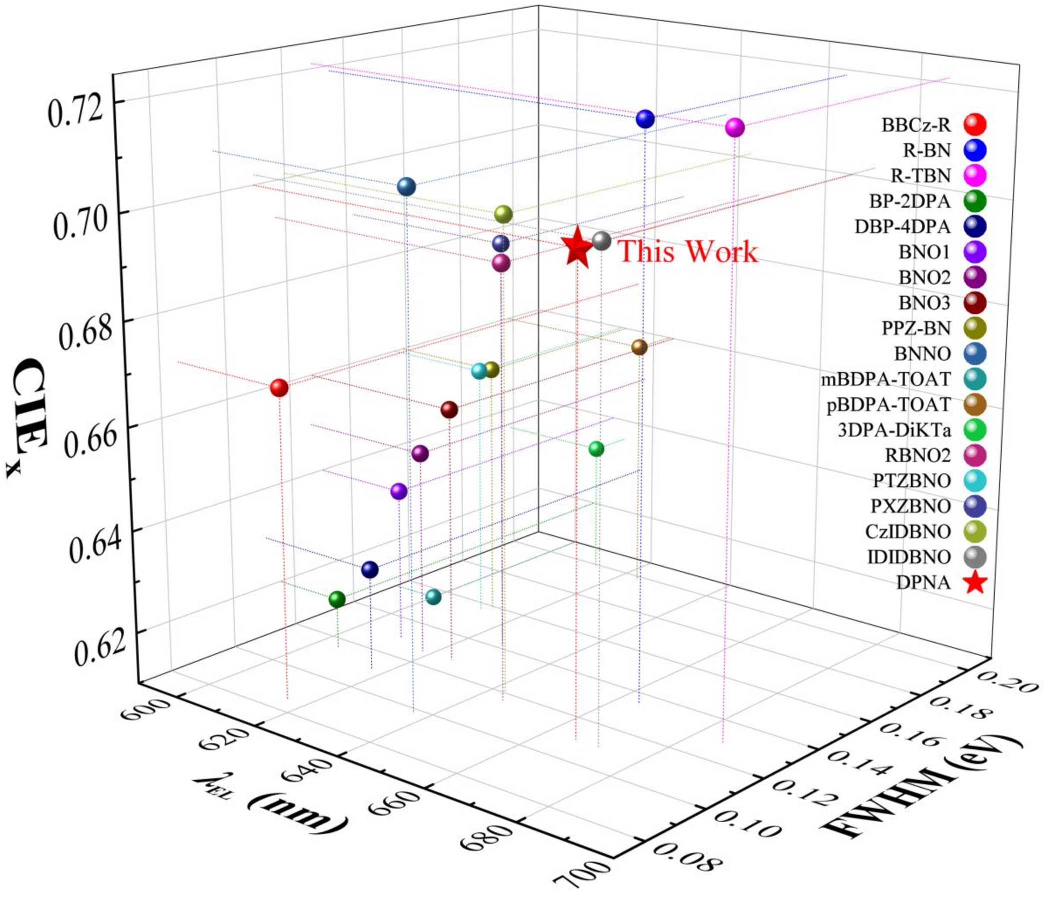

Figure S31. CIE_x (The Commission Internationale de l'Éclairage of x value), λ_{EL} (the peak wavelength of the electroluminescence in nm), and FWHM (full width at half maximum in eV) of this work compared to the reported red color MR emitters. The molecular structures of BBCz-R,[3] R-BN and R-TBN,[4] BP-2DPA and DBP-4DPA,[5]

BNO1-BNO3,[6] PPZ-BN,[7] BNNO,[8] mBDPA-TOAT and pBDPA-TOAT,[9] 3DPA-DiKTa,[10] RBNO2,[11] PTZBNO and PXZBNO,[12] CzIDBNO and IDIDBNO,[13] can refer to Figure S19.

Table S5. The comparison EL Performances of the red color approaching BT. 2020 gamut.

Emitter	EL(nm)	FWHM (nm/eV)	CIE_x	Ref.
BBCz-R	616	26/0.08	0.67	3
R-BN	664	48/0.13	0.72	4
R-TBN	686	49/0.13	0.72	
BP-2DPA	605	34/0.12	0.62	5
DBP-4DPA	617	34/0.11	0.63	
BNO1	610	39/0.13	0.64	6
BNO2	618	39/0.13	0.65	
BNO3	625	40/0.13	0.66	
PPZ-BN	613	48/0.16	0.66	7
BNNO	637	32/0.10	0.71	8
mBDPA-TOAT	600	45/0.16	0.61	9
pBDPA-TOAT	624	62/0.20	0.66	
3DPA-DiKTa	613	60/0.20	0.64	10
RBNO2	645	39/0.12	0.69	11
PTZBNO	612	47/0.16	0.66	12
PXZBNO	627	45/0.14	0.69	
CzIDBNO	643	38/0.12	0.70	13
IDIDBNO	671	39/0.11	0.70	
DPNA	666	40/0.11	0.70	This work
DPNA-F	661	40/0.11	0.70	
DPNA-tBu	667	40/0.11	0.71	

References

(1) H. Joshi; F. S. Kamounah; G. van der Zwan; C. Gooijer; L. Antonov. *Journal of the Chemical Society, Perkin Transactions 2* **2001**, (12), 2303-2308.

(2) W.-C. Chen; I. Chao. *Physical Chemistry Chemical Physics* **2021**, *23* (5), 3256-3266. O. López-Estrada; H. G. Laguna; C. Barrueta-Flores; C. Amador-Bedolla. *ACS Omega* **2018**, *3* (2), 2130-2140. H.-S. Ren; M.-J. Ming; J.-Y. Ma; X.-Y. Li. *The Journal of Physical Chemistry A* **2013**, *117* (33), 8017-8025.

(3) M. Yang; I. S. Park; T. Yasuda. *J Am Chem Soc* **2020**, *142* (46), 19468-19472.

(4) Y. Zhang; D. Zhang; T. Huang; A. J. Gillett; Y. Liu; et al. *Angew Chem Int Ed Engl* **2021**, *60* (37), 20498-20503.

(5) K. R. Naveen; S. J. Hwang; H. Lee; J. H. Kwon. *Advanced Electronic Materials* **2021**, 2101114.

(6) Y. Zou; J. Hu; M. Yu; J. Miao; Z. Xie; et al. *Adv Mater* **2022**, e2201442.

(7) H. Chen; T. Fan; G. Zhao; D. Zhang; G. Li; et al. *Angewandte Chemie International Edition* **2023**, *62* (20), e202300934.

(8) T. Fan; M. Du; X. Jia; L. Wang; Z. Yin; et al. *Adv Mater* **2023**, *35*, e2301018.

(9) X. C. Fan; K. Wang; Y. Z. Shi; J. X. Chen; F. Huang; et al. *Advanced Optical Materials* **2021**, *10* (3), 2101789.

(10) S. Wu; A. Kumar Gupta; K. Yoshida; J. Gong; D. Hall; et al. *Angewandte Chemie International Edition* **2022**, *61* (52), e202213697.

(11) Y. Zou; J. He; N. Li; Y. Hu; S. Luo; et al. *Mater Horiz* **2023**, *10* (9), 3712-3718.

(12) J. He; Y. Xu; S. Luo; J. Miao; X. Cao; et al. *Chemical Engineering Journal* **2023**, *471*, 144565.

(13) N. L. an-Yun Jing, Xiaosong Cao, Han Wu, Jingsheng Miao, Zhanxiang Chen, Manli Huang, Xinzhong Wang, Yuxuan Hu, Yang Zou, Chuluo Yang. *Sci. Adv.* **2023**, *9* (30), eadh8296
